# Ethnobotanical uses in the Ancona district (Marche region, Central Italy)

**DOI:** 10.1186/s13002-019-0288-1

**Published:** 2019-02-05

**Authors:** Lara Lucchetti, Silvia Zitti, Fabio Taffetani

**Affiliations:** Department of Agricultural, Food and Environmental Sciences, Marche Polytechnic University, via Brecce Bianche, 60131 Ancona, Italy

**Keywords:** Ethnobotany, Traditional local knowledge, Wild plant uses, Marche region, Italy

## Abstract

**Background:**

The study is a survey of the traditional uses of plants in the Ancona district, in the Marche region, Central Italy.

**Methods:**

The information derives from ethnobotanical investigations conducted with an open questionnaire among the rural population in three areas of the Ancona district that are representative of the socio-economic and environmental assets of the entire district: the Mount Conero area on the Adriatic coast; the municipality of Osimo, as an inland hilly area; and the ‘Gola della Rossa–Frasassi’ area, in the Apennines.

**Results:**

A total of 120 informants cited 195 species. The ethnobotanical data concern medicinal (122 species), food (119), veterinary (53), superstitious/religious (61), cosmetic (30), domestic (27), dyeing (17), recreational (17), repellent (15), craft (10), and miscellaneous (29) uses, along with inclusion in local sayings and proverbs (25). The species with the greatest number of categories of use here was *Sambucus nigra* L. Among the other species with the greatest numbers of categories of use, there were *Matricharia chamomilla* L., *Salvia officinalis* L., *Urtica dioica* L., *Papaver roheas* L., and *Rosa canina* L. For each use, comparisons with national and regional literature were made.

**Conclusions:**

Some uses are commonly known across the three areas; others are sectoral and are new for the Marche region. The survey increases our present-day knowledge of the traditional local uses of plants in the Marche region, in terms of medicinal and food uses, and of ethnobotanical aspects as a whole, which will allow many of these uses to be preserved in the future.

## Background

The use of wild plants in Italian rural communities was a common practice, especially in the traditional sharecropping rural society of Central Italy that was largely based on self-sufficiency through self-consumption [1]. In this kind of society, in addition to the most common kind of uses as medicines and food, a lot of plants were used for many different aspects of daily life, such as craft work and home tools. However, the rural culture that included the knowledge of the use of spontaneous plants began to fragment from the second half of the twentieth century due to the progressive depletion of the population of the countryside, and to urbanisation and widespread industrialisation [2–4]. The use of sharecropping contracts ended in 1964 also in the Marche region, and this led to changes in the production structure, with the spread of large-scale agriculture that was disconnected from the territory itself. This contributed to the loss of identity of the rural society, and of its knowledge and traditions. Research such as the present study can contribute to the conservation of the knowledge related to traditional practices, which are now fragmented and remain almost exclusively with older people [5].

The present study collected and analysed the knowledge of ethnobotanical uses that are still widespread in the Ancona district, and considers not only medicinal and food uses, but also veterinary, superstitious/religious, cosmetic, domestic, dyeing, recreational and repellent uses, and craft uses for wood, and cases where plants are mentioned in sayings and proverbs. Three areas among the rural populations of the Ancona district that are representative of the socio-economic and environmental assets of the entire district were chosen for this study: the Mount Conero area on the Adriatic coast; the municipality of Osimo, as an inland hilly area; and the Gola della Rossa–Frasassi area, in the Apennines.

The aims were thus to

- Collect the traditional knowledge about wild plant uses that still remains in the population of central Marche;
- Compare data collected with the literature on regional and national ethnobotanical surveys;
- Identify new uses according to the Ancona district.

## Methods

### Survey areas

**Fig. 1:**
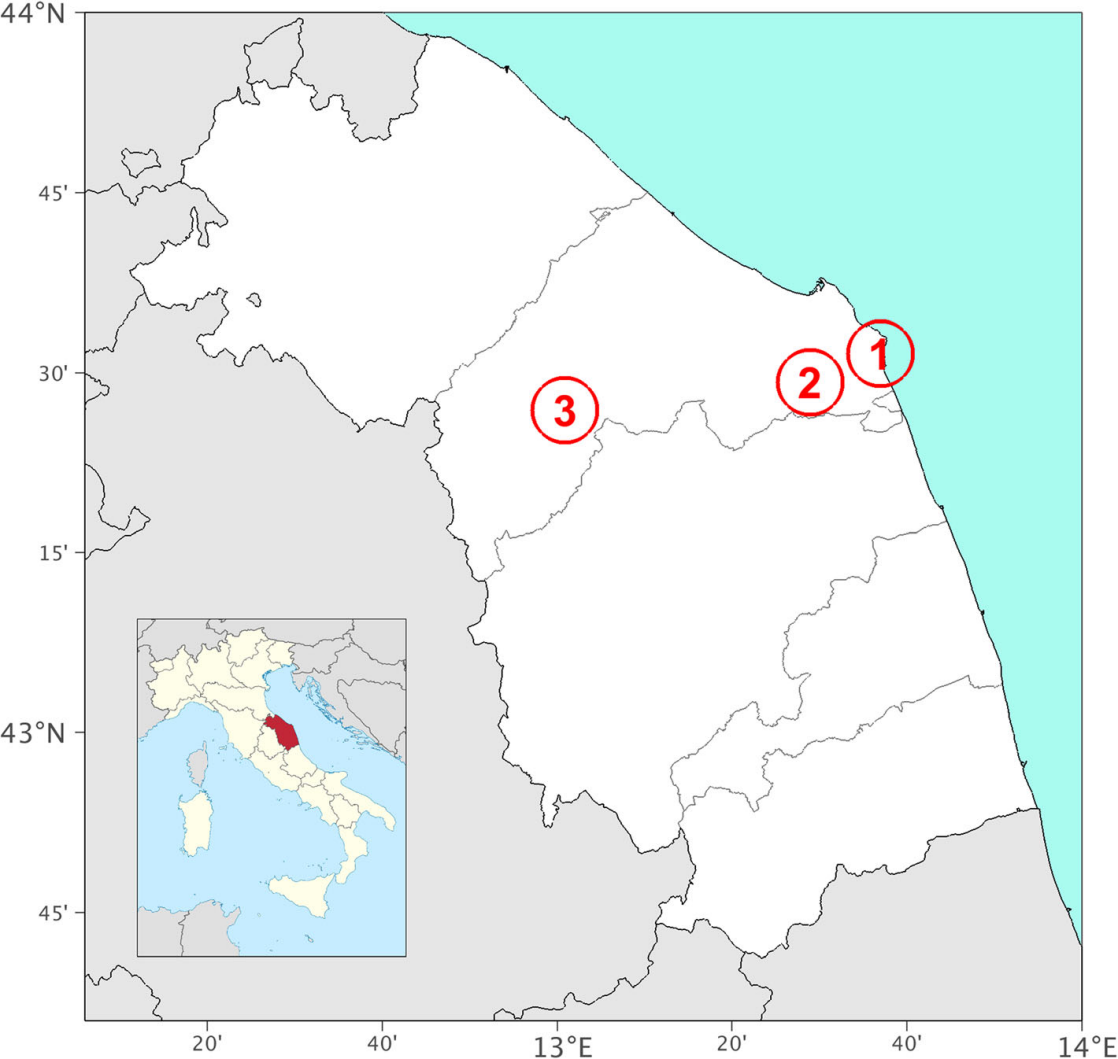
Map showing the three study areas: 1, Mount Conero area; 2, Osimo area; 3, Gola della Rossa–Frasassi area

These ethnobotanical studies were conducted in three different areas in the Ancona district (Marche region, Central Italy). The Ancona district is one of the five provinces of the Marche region, and it includes a small area of the Apennines (34%), and wider hilly inland areas with flat stretches and an extended coastline, which together account for the remaining two thirds of the territory [6, 7]. The three areas of this study were thus designed to fall into each of these three sectors: the Mount Conero area on the Adriatic coast; the inner hilly area of the municipality of Osimo; and the Apennine area of Gola della Rossa–Frasassi (Fig. 1).

#### The area of Conero Park

The Mount Conero area extends along a coastal strip in a central position of the Marche region, and it includes part of the municipalities of Ancona, Camerano, Sirolo, and Numana. On the basis of the bioclimatic indices of Rivas-Martinez et al. [8], the territory of Mount Conero belongs to the Mediterranean macrobioclimate, with a pluviseasonal oceanic climate, upper meso-Mediterranean thermotype, and low subhumid ombrotype [9]. The territory is mainly hilly, and Mount Conero is the highest peak (572 m a.s.l.). Thirteen percent of the territory is urbanised [10], and 50% is dedicated to agriculture [11]. The economic enterprises are mostly tourism and manufacturing [12].

This area includes the Mount Conero Regional Natural Park (Parco Naturale Regionale del Conero), which covers a total area of 5914 ha, and is characterised by different habitats of high floristic and geological value. These include three Sites of Community Importance (SCI) and one Zone of Special Protection (ZSP). The prevailing plant landscape in the central core of Mount Conero is constituted by woods of evergreen sclerophylls that alternate with reforestation with conifers and deciduous forests. Along the cliffs above the sea, there is rupiculous vegetation and Mediterranean scrubland. The more internal hilly areas mainly comprise agricultural landscapes that are mixed with oak woods (*Quercus pubescens* Willd.), hygrophilous vegetation along the water courses, and broom shrubs (*Spartium junceum* L.) that colonise the abandoned fields. The flora includes 1169 entities [9], some of which here reach the northern limits of their distribution along the western Adriatic coast, including *Ampelodesmos mauritanicus* (Poir.) T. Durand and Schinz, *Coronilla valentina* L. and *Euphorbia dendroides* L. In this area, the ethnobotanical surveys were conducted in the municipalities of Camerano, Sirolo (a hamlet of San Lorenzo, Coppo) and Numana, and in the hamlets of Poggio and Massignano in the municipality of Ancona.

#### The area of the municipality of Osimo

The municipality of Osimo extends over 10,600 ha, and the territory is mainly hilly (highest peak, Monte della Crescia, 361 m a.s.l.), and it alternates with valleys near the Musone River. The macrobioclimate is temperate with a sub-Mediterranean variant, lower mesotemperate thermotype, and lower humid ombrotype [8]. This territory is predominantly agricultural, with marginal environments that are characterised by natural and semi-natural vegetation, with some residual woods that were the subject of recent studies [13, 14], and some riparian areas. Osimo has a population of 34,918 inhabitants (ISTAT 2017) and is classified as ‘level 2’ in terms of its degree of urbanisation (ISTAT 1 January 2018) [15]. The local enterprises are mainly based on manufacturing [12], and cultivation covers 7310 ha. In this area, the ethnobotanical surveys were conducted in the hamlets of Campocavallo, Passatempo, San Sabino, Padiglione, San Paterniano, and San Biagio.

#### The area of Gola della Rossa–Frasassi

The third area is located in the mountain sector of the province of Ancona, and it falls partly within the Regional Natural Park of the Gola della Rossa–Frasassi. The territory is mainly mountainous (highest peak, 1093 m a.s.l.) and consists of the two limestone gorges ‘Gola di Frasassi’ and ‘Gola della Rossa’, and includes also Scappuccia Valley and Valdicastro Valley. The bioclimate is temperate of the sub-Mediterranean variant, upper mesotemperate thermotype, and lower humid ombrotype [8]. The vegetation of the mountain areas is mainly mixed deciduous forests that are dominated by hornbeam and flowering ash, and at higher altitudes, beech and grasslands with shrubs. The calcareous gorges with southern exposure host Mediterranean sclerophyllous woods and rupiculous vegetation, with the presence of endemic species, including *Moehringia papulosa* Bertol., which is endemic to the Marche gorges [7]. In the lower areas of the valleys, the landscape is agricultural, with cultivated fields alternating with small residual woody nuclei, with hedges, shrubland, and margin vegetation. The population is mainly concentrated in the urban centres of the park, with the production activities located at the bottoms of the valleys, as relatively fragmented agricultural activities. In the area of Gola della Rossa–Frasassi, the ethnobotanical surveys were conducted in the hamlets of Castellaro, Trivio, Forchiusa, Serralta, Sasso, Montirone, and Sant’Elena, and in the municipality of Serra San Quirico.

### Ethnobotanical research methods

The ethnobotanical surveys were conducted in the small towns and rural villages of the three areas in the Ancona district between 2008 and 2011, and involved a total of 120 people, defined as the ‘informants’. These informants were not chosen completely at random within the territories, but were chosen through selection of individuals who according to their ages (more than 50 years of age) or cultural or social backgrounds would have knowledge of the plant uses, either directly or as passed down by their families. This was achieved by means of word of mouth from some known contacts to identify relevant informants, using the ‘snow-ball sampling’ method [16].

The informants were initially introduced to the aims and methods of the interviews, and then asked for their consent to proceed. Before proceeding with the interviews, it was ascertained that the informants were native to the particular survey area, in terms of being born and raised there. During the interviews, the informants were asked open questions, such as “Which plants were used, and for what use? How were these plants used, who collected them, and where and when? Were there sayings or proverbs related to any specific plants?” Data were also collected on the informants, in an anonymous form, as year of birth, initials of name and surname, gender, level of education, and work activity. Italian was used as the language of the interviews. Table 1 includes the local names of the plants that were collected, where sometimes the local names were different across the three study areas.

**Table 1:** The species of ethnobotanical interest in the Ancona district

Scientific name	Family	Local names	Parts used	Uses	References for similar uses
*Acer campestre* L.	*Sapindaceae*		Wood	Craft: handles, tools [37]	
			Whole plant	Mix: supports for grapevine [37]	
*Achillea collina* (Becker ex Rchb.f.) Heimerl	*Asteraceae*	*Millefoje, stagnasangue (g)*	Flower	Food: **fried flower in salted batter**	
			Leaves	Med: infusion as cicatrizer [27]	
			Aerial part	Sup/rel: **stems in pocket, against haemorrhoids**	
*Adonis annua* L. ssp*. cupaniana* (Guss.) C. Steinberg	*Ranunculaceae*		Leaves, flowers	Med: infusion as diuretic [37]	
*Aesculus hippocastanum* L*.*	*Sapindaceae*	*Castagna selvatica*	Fruit	Sup/rel: under the pillow against colds [37]	
*Agrimonia eupatoria* L*.*	*Rosaceae*	*Erba de andata (o)*	Leaves	Med**: leaf infusion as digestive**	
				Food: **leaves for filling fresh pasta**	
*Ailanthus altissima* (Mill.) Swingle	*Simaroubaceae*		Leaves	Med: leaf infusion as anti-diarrhoea	Similar use of bark in [27]
				Vet: **for feeding silkworms**	
			Wood	Craft: **handles, tools** Dom: **firewood**	
*Alliaria petiolata* (M.Bieb.) Cavara and Grande	*Brassicaceae*	*Agliaria (o), erba aglina (g)*	Leaves, flowers	Med: infusion to treat cough [27]	
			Leaves	Food: to flavour salads [30, 34], roasted meat; piadina filling	To flavour various dish in [27]
				Vet: **in dairy cow feed**	
*Allium cepa* L*.*	*Amaryllidaceae*		Bulb	Med: fresh bulb cut in half rubbed on the skin as disinfectant to heal insects bites [23, 26]	
				Sup/rel: bulbs cut in half with spoonful of coarse salt on top to predict the weather [23]	
*Allium neapolitanum* Cirillo	*Amaryllidaceae*	*Cipollotto del diavolo (o)*	Bulb	Med: raw bulbs eaten as vermifuge	Similar use of *Allium sativum* L. in [4, 21, 37]
				Food: raw in salads [34]	
				Vet: **bulbs macerated in wine to heal rabies in dogs**	
				Sup/rel: bulbs in necklaces to protect against devil’s eye	Similar use of. *Allium sativum* L. in [21, 37]
				Rep: **bulbs macerated in water against aphids**	
			Flowers	Food: sautéed flowers to season pasta	
				Dom: **flowers used in floral decorations**	
*Allium sativum* L.	*Amaryllidaceae*		Bulb	Med: one raw bulb or four bulbs boiled in milk and eaten to heal intestinal warms [23, 33]; one bulb under the pillow to heal intestinal warms in children [4, 23]; bulb poultice with olive oil or beeswax to heal calluses [23, 26]; rubbed fresh bulb to heal insects bites [21, 26]	
				Prov: ‘*se voi l’aio grosso, a Natale lo devi avè posto*’	
*Aloysia citriodora* Palau*.*	*Verbenaceae*	*Cedrina (g)*	Leaves	Cosm: leaves in bath water to perfume the skin [23]	
				Dom: **dry flowers in floral decorations**	
*Amaranthus retroflexus* L.	*Amaranthaceae*		Flowers	Dom: **dry flowers in floral decorations**	
*Ampelodesmos mauritanicus* (Poir.) T.Durand and Schinz	*Poaceae*	*Saracco (c)*	Leaves	Mix: leaves used to make string and rope [22]	
*Anagallis arvensis* L*.*	*Primulaceae*	*Centocchio (o)*	Aerial part	Med: decoction of aerial part to heal cough [27]	
				Vet: aerial parts with leaves of *Urtica dioica* L. and dry bread for feeding laying hens [37]	
*Apium graveolens* L*.*	*Apiaceae*	*Acquaiola (o)*	Aerial part	Med: infusion of aerial part as digestive and diuretic [30]; leaf pack as emollient*	*Similar use against bruises [37] or to treat chilblains [23]
				Cosm: leaf pack to treat dry skin	Similar use for healing skin complaints and chilblains [23, 37]
				Sup/rel: fresh plant eaten as aphrodisiac; against devil’s eye [30]	
*Arbutus unedo* L.	*Ericaceae*		Fruits	Food: fruit eaten raw or preserved in alcohol to make a liquor [4, 21, 27]	
*Arctium minus* (Hill) Bernh.	*Asteraceae*		Leaves	Med: leaves in pack on feet as diaphoretic to heal bronchial diseases (correlated to fever) [36]	
				Cosm: leaf juice rubbed on scalp to heal dandruff; leaf decoction to heal acne	Similar use to heal hair loss [25]
			Stems	Food: ***boiled stems as side dish*** [36, 38]	
*Artemisia vulgaris* L*.*	*Asteraceae*	*Erba di S. Giovanni (g)*	Leaves	Med: leaf infusion to regularise menstruation [37]	
				Food: ***some raw leaves in salads***	similar uses in soups [37] and for *Artemisia absinthium* L. [30])
				Sup/rel: **on St. John’s night, stems of** ***Artemisia vulgaris*** **L*., Ruta graveolens*** **L*., Rosmarinus officinalis*** **L., and** ***Lavandula*** **sp. in the pocket or under the pillow to protect against witches; protect during a travel**	
				Rep: **leaves macerated in water against plant caterpillars**	
*Arum italicum* Mill.	*Araceae*	*Erba biscia (o)*	Leaves	Med: leaves applied as antirheumatic [37]	
				Vet: leaf decoction as diuretic for pigs	Roots as feeding for pigs [21]
				Dom: boiled leaves for washing clothes, pots [37]	
			Whole plant	Sup/rel: **plant brings bad luck**	
*Arundo donax* L.	*Poaceae*	*Canna (o, c, g)*	Leaves	Med: leaf infusion as diuretic [37]	
				Mix: dry leaves smoked as tobacco substitute [4]	
			Twigs	Sup/rel: *Arundo donax* L. and *Olea europaea* L*.* twigs to make a cross to protect fields [23]	
				Craft: **to make a support for knitting pins**, to make ‘*raganella*’ [37]	
				Recr: to make whistles [37]	
				Mix: to support plants in the orchards, to make baskets [37]	
*Asparagus acutifolius* L.	*Asparagaceae*	*Sparaghi (c), asparagina (c, g)*	Shoots	Med: eat boiled shoots as diuretic [4, 30]; shoots decoction together with *Elymus repens* (L.) Gould. as diuretic	
				Food: boiled shoots as side dish [30], seasoning for risotto and omelettes [21, 30], [4, 41, 44, 48]	
				Dye**: boiling water used to dye fishing nets green**	
			Aerial part	Dom: dry plants used in floral decorations [36]	
*Avena sativa* L*.*	*Poaceae*	*Venella (g)*	Seeds	Med: infusion and wraps to heal rheumatic pain [37]	
			Aerial part	Vet: dry plants to feed rabbits, horses, cattle [36]	
			Ears	Recr: ears pulled by girls and boys, and counted to forecast number of children or husbands [37]	
*Barbarea vulgaris* R. Br*.*	*Brassicaceae*	*Crescione (g)*	Leaves	Food: ***raw leaves in salads***	In soups [30, 44]
*Bellis perennis* L.	*Asteraceae*	*Pasquetta (o), margherita (g)*	Leaves	Med: raw leaves eaten as depurative [4]; wrap of raw leaves to treat sores [37]	
				Food: ***raw leaves in salads*** [4, 39, 42]***; in soups*** [39, 41]	
			Flowers	Sup/rel: *infiorata* [4]	
				Recr: flowers used to make necklaces and for ‘*m’ama non m’ama*’ game [37]	
*Borago officinalis* L.	*Boraginaceae*	*Boraggine, borragine (c, o, g), borragia (g)*	Leaves	Med: leaf infusion to heal cough [25, 31] as depurative [25]; leaf wraps to heal sores and reddened skin*	*Emollient in [30]
				Food: leaves raw in salads [27], boiled as side dish [41, 44], seasoning for pasta and risotto [4, 44], filling for fresh pasta or pies [4, 21, 42], soups [4, 21, 27, 41, 45], omelettes [27, 41, 42], fried [4, 21, 44], fried with mozzarella and anchovy rolls	
				Cosm: leaves in bath water to clean skin	Emollient properties in [30, 43]
			Flowers	Food: flower used to flavour vinegars*; in fresh salads	*Leaves used to flavour wine [25]
				Dye: flowers used to dye clothes blue; colour is strongest if flowers are just harvested [37]	
*Brassica oleracea* L*.*	*Brassicaceae*	*Cavolo, verza (g)*	Leaves	Med: fresh leaves used to make wraps to heal rheumatic pain [4, 26, 31]	
				Vet: fresh leaves used to make wraps to heal bruises [37]	
*Calendula officinalis* L*.*	*Asteraceae*	*Calenda (o, g)*	Flowers	Med: macerated flowers in the wine used to heal chilblains; ointment with olive oil and flowers used as emollient [26]; ointment with flowers used as cicatrizer	The use is similar to the lenitive one and to heal rheumatic pains in [26, 33, 43]
				Food: **flowers for seasoning risotto**	
				Sup/rel: flowers used in ‘*infiorata*’ [37]	
*Calepina irregularis* (Asso) Thell.	*Brassicaceae*	*Erba del tacchì (o)*	Leaves	Food: ***leaves boiled to make omelettes***	In soups [39]
			Whole plant	Sup/rel: **brings good luck**	
			Flowers	Mix: **flowers used to decorate churches for marriages**	
*Calystegia sepium* (L.) R.Br.	*Convolvulaceae*	*Campanella (o)*	Leaves	Med: leaf decoction used as laxative [25, 27]	
			Flowers	Mix: **flowers used in wedding bouquets**	
*Campanula rapunculus* L.	*Campanulaceae*	*Lattughella (g)*	Leaves	Food: raw leaves in salads [4, 21, 24, 39]	
*Cannabis sativa* L*.*	*Cannabaceae*	*Canapa (c)*	Aerial part, stems	Mix: to make string, cord [23]	
*Capsella bursa pastoris* (L.) Medik.	*Brassicaceae*	*Cimino (o)*	Leaves	Med: leaf decoction to heal menstrual pain [25]	
				Food: ***raw leaves in salads or boiled in vegetable mixtures as side dish*** [4, 39]	
			Whole plant	Sup/rel: **brings good luck**	
*Carex pendula* Huds*.*	*Cyperaceae*	*Cannucciaia*	Stems	Mix: stems used to make seats for straw chairs [36]	
*Castanea sativa* Mill*.*	*Fagaceae*	*Castagna (g)*	Fruits	Food: fruit frequently eaten, roasted, cooked under ashes, boiled with laurel leaves; flour used to make bread and cakes (‘*castagnaccio*’) [21]	
*Celtis australis* L.	*Cannabaceae*	*Olmo bianco (o), spaccasassi (g)*	Leaves	Med: leaf decoction as anti-inflammatory of oral cavity [31]	
				Vet: **leaves for feeding the cattle**	
			Fruits	Food: **fruit used for flavouring grappa**	
				Sup/rel: fruit used for making rosaries	
				Recr: fruit used to make necklaces; fruit used with blowpipes [36]	
*Ceratonia siliqua* L*.*	*Fabaceae*	*Carruba, carrobie (c)*	Seeds	Food: ***seeds eaten as sweets or used to make sweets with onion*** [35, 48]	
			Twigs	Mix: **young twigs to make ties**	
*Cercis siliquastrum* L*.*	*Fabaceae*		Flowers	Food: ***flowers fried in sweet batter*** [37]	
*Chelidonium majus* L*.*	*Papaveraceae*		Latex	Med: latex used as cicatrizer [31]; latex dissolved in water for internal use to heal heartburn [25]	
			Aerial part	Dye: plant used to dye clothes yellow [37]	
*Chenopodium album* L*.*	*Amaranthaceae*	*Spinacio selvatico (g)*	Leaves	Food: ***leaves boiled and served as side dish, like spinach*** [39, 41]	
*Chenopodium bonus-henricus* L*.*	*Amaranthaceae*	*Buon enrico, spinacio selvatico (g)*	Leaves	Med: boiled leaves put on burns as emollient	Similar use in [37]
				Food: boiled leaves in vegetable mixtures, for seasoning risotto, filling fresh pasta; raw leaves with pine nuts, walnuts, oil; boiled as seasoning [39, 48]	
*Cichorium intybus* L*.*	*Asteraceae*	*Grugni (c, g), grugni selvatici, grugni campagnoli (g)*	Leaves	Med: leaves decoction as depurative and diuretic [21, 43]; as anti-anaemic [23];	
				Food: raw young leaves in salads [4, 39, 41, 42, 45], boiled in vegetable mixture as a side dish [4, 21, 39, 41, 44, 45], boiled to fill fresh pasta [21], ***boiled and preserved in oil*** [48]	
				Vet: leaves for feeding rabbits **to heal intestinal worms**	
			Roots	Food: roasted roots as surrogate for coffee [37]	
				Sup/rel: **roots have protective value**	
			Whole plant	**Dye: to dye clothes in yellow**	
*Cirsium arvense* (L.) Scop*.*	*Asteraceae*		Roots	Med: chew raw roots against toothache [37]	
			Leaves	Food: ***leaves boiled and sautéed as side dish*** [39]	
*Citrus limon* (L.) Osbeck	*Rutaceae*		Flowers	Med: flowers decoction to heal cough [37]	
				Cosm: flowers decoction to treat oily skin	Fruits used to heal skin disease [26]
				Dom: flowers used to perfume rooms and surroundings [37]	
			Fruits	Dom: fruit juice used with salt and vinegar to clean pots [37]	
*Clematis vitalba* L*.*	*Ranunculaceae*	*Vitalbe, vitalbene, vitarvene (c), barba dei frati, barba dei vecchi, vitalla (g)*	Leaves	Med: leaf decoction as diuretic [37]	
				Mix: dry leaves smoked as tobacco substitute [37]	
			Shoots	Food: boiled young shoots as side dish [39], to season risotto, to make omelettes [4, 39, 41, 44, 45], to preserve in oil	
			Stems	Mix: young stems used to make string [4, 37]	
			Flowers	Dom: flowers used in flora decorations [36]	
*Clinopodium nepeta* (L.) Kuntze*.*	*Lamiaceae*	*Mentuccia (c, o, g), menta (o, g), menta selvatica (g)*	Leaves, flowers	Med: poultice of leaves as emollient [27, 37]	
				Food: leaves used to flavour meat, vegetables, omelettes, soups [4, 34, 39, 41, 44]	
				Cosm: **leaves chewed to heal bad breathe**	
			Whole plant	Prov: ‘*Chi vede la mentuccia e non ne sente l’odore non vede la Madonna quando muore*’	
*Convolvulus arvensis* L*.*	*Convolvulaceae*	*Campanelle (g)*	Leaves	Med: crushed fresh leaves applied to skin to heal pimples [37]	
			Flower	Food**: flowers sucked as snack**	
*Cornus mas* L*.*	*Cornaceae*	*Grugnale (o, g)*	Shoots	Med: shoot infusion as febrifuge [37]	
			Fruits	Food: fruit used to flavour grappa [23, 42]; fruit eaten raw [37, 42, 45]	
			Flowers	Cosm: **flowers decoction to heal oily skin**	
			Wood	Craft: **wood used to build boats**	
				Prov: ‘*Sei un grugnale*’	
*Cornus sanguinea* L*.*	*Cornaceae*	*Sanguinella (g)*	Wood	Craft: handles, tools [37]	
*Corylus avellana* L*.*	*Betulaceae*		Fruits	Food: fruit eaten fresh or to make cakes [37, 41, 42]	
			Whole plant	Sup/rel: **plant protects against lightning**	
*Cota tinctoria* (L.) J.Gay*.*	*Asteraceae*	*Falsa camomilla, camomilla tinta (g)*	Flowers	Sup/rel: **flowers used in ‘*infiorata*’**	
				Dye: flowers in boiled water to dye wool yellow [37]	
*Crataegus monogyna* Jacq*.*	*Rosaceae*	*Biancospino, porcospino, albero delle Perelle (g)*	Leaves, flowers	Med: flowers and leaf infusion to heal heart problems, as anti-hypertensive [21, 23, 42]	
			Fruits	Med: **dry fruit heated in little bag and used to heal rheumatic pains**	
				Food: fruit eaten raw, to make jams, liqueurs [37, 41, 42]	
				Vet: **fruit poultice used to heal ‘*spallone*’ in cattle** (bruising caused by ‘giogo’-yoke)	
			Wood	Dom: wood used to light fires and heat the oven, with *Olea europaea* L*.* branches. It was said to give bread a good aroma [36]	
				Sup/rel: plant had religious value, because it flowered from the stick of Giuseppe d’Arimatea	Other magic uses in [37]
*Crepis vesicaria* L*.*	*Asteraceae*	*Grugno porcino (g)*	Basal rosette	Food: leaves boiled in vegetable mixture as side dish [4, 34, 39, 41, 44]	
*Crithmum maritimum* L*.*	*Apiaceae*	*Paccasassi, spaccasassi (c)*	Leaves, shoots	Food: leaves boiled in water and vinegar and preserved in olive oil [24, 39, 48]	
*Cruciata laevipes* Opiz	*Rubiaceae*	*Erba croce (o)*	Leaves	Med: leaf juice drank as vermifuge* [37], **leaf decoction to heal intestinal obstructions**	
			Roots	Dye**: roots used to dye wool red**	
*Cydonia oblonga* Mill*.*	*Rosaceae*		Fruits	Food: fruit used to make jams [37, 41], sometimes with grape berries	
				Dom: some fruits put in fruit basket to perfume the kitchen [4, 37]	
*Cynodon dactylon* (L.) Pers*.*	*Poaceae*	*Gramaccia (c, g)*	Roots	Food: ***raw roots eaten in salads*** [5]	
			Aerial part	Vet: plant really liked by pigs	Veterinary food use for ruminants and horses [4]
			Plant	Prov: ‘*Essere cattivo come la gramigna*’	
*Daucus carota* L*.*	*Apiaceae*		Roots	Med: roots crushed and poultice, used to heal burns [26, 27]	
				Food: roots eaten and boiled as side dish in famine period [23, 39]	
			Stems	Mix: stems used to tie sheaves [36]	
*Dioscorea communis* (L.) *Caddick and Wilkin*	*Dioscoreaceae*	*Viticella (g)*	Shoots	Food: shoots boiled and used to make omelettes [24, 39, 44]	
*Diplotaxis erucoides* (L.) DC.	*Brassicaceae*	*Rughetta (o), fiore bianco (c), carrugola selvatica, carrugola, carrucola (g)*	Leaves	Med**: raw leaves eaten as digestive**	
				Food: raw leaves in salads; boiled as side dish [34, 39, 41, 44]	
*Diplotaxis tenuifolia* (L.) DC*.*	*Brassicaceae*		Leaves	Med: **raw leaves eaten as digestive**	
				Food: raw leaves for seasoning pizza, salads; boiled for seasoning pasta [4, 34, 39, 41, 42, 45]	
*Echium vulgare* L*.*	*Boraginaceae*	*Erba viperina (g)*	Leaves	Food: ***leaves of basal rosette boiled in vegetable mixtures as side dish*** [39, 44]	
*Elymus repens* (L.) Gould*.*	*Poaceae*	*Gramaccia (c, g); gramigna, grano delle formiche (o)*	Roots	Med: root decoction as depurative [4, 31, 43, 44]	
			Seeds	Food: **seeds used for flavouring bread**	
			Ears	Recr: **children play with ears, detaching them one by one to see if desire comes true**	
			Aerial part	Med: decoction to heal abdominal pain; **crushed plant put on forehead to heal nose bleed**	
			Whole plant	Prov: ‘*Le donne molto feconde sono come la gramaccia*’, ‘*Esse taccati come la gramigna*’	
*Equisetum arvense* L*.*	*Equisetaceae*	*Coda cavallina (c)*	Aerial part	Med: stem decoction used as footbath to heal excessive perspiration [4]	
			Shoots	Food: young shoots fried or boiled to make omelettes [37, 44, 45]	
*Equisetum telmateia* Ehrh*.*	*Equisetaceae*	*Coda cavallina (g)*	Aerial part	Med: stem decoction used as footbath to heal excessive perspiration [4]; stem decoction instilled in nose to heal nosebleed [26] or inhaled against nosebleed	
				Cosm: to reinforce nails, fingers were put in stem decoction [26]. Stem decoction used to purify skin [36]	
				Dom: stems used to polish kitchenware [23]	
			Shoots	Food: young shoots fried or boiled to make omelettes [37, 44, 45]	
*Eucalyptus camaldulensis* Dehnh*.*	*Myrtaceae*	*Ocalitto (o)*	Leaves	Med: leaf decoction as antipyretic [37]	
				Food: ***leaves used to flavour grappa***	Similar use for *E*. *globolus* Labill. [36]
				Vet: leaves rubbed on animals to heal parasites	Similar use for *E*. *globolus* Labill. [36]
				Dom: flowers, fruit, and twigs used in floral decorations [36]	
				Rep: leaves used in the house against anopheles [37]	
*Euonymus europaeus* L*.*	*Celastraceae*		Wood	Craft: wood used to make spindles [37]	
*Euphorbia helioscopia* L*.*	*Euphorbiaceae*	*Latte del diavolo (o)*	Latex	Sup/rel: **latex has protective value**	
*Euphorbia lathyris* L*.*	*Euphorbiaceae*		Whole plant	Rep: species planted in orchards to kept them clear from rats [24]	
*Euphorbia peplus* L*.*	*Euphorbiaceae*	*Tortumaio (c)*	Latex	Med: fresh latex on wounds as cicatrizer	To heal warts in [26]
*Ficaria verna* Huds*.*	*Ranunculaceae*	*Botton d’oro (g)*	Leaves	Med: **crushed leaves to heal arthritis pain**	
*Ficus carica* L*.*	*Moraceae*	*Figo (o, c)*	Latex	Med: latex used to heal warts and calluses [4, 21, 26, 37]	
				Cosm: **latex appears to be used to be more tanned**	
			Fruits	Fruits are eaten raw or used to make jams [21, 41, 42, 45]	
			Shoots, twigs	Sup/rel: shoots put in St. John’s water [37]	
			Twigs	Sup/rel: **twigs used to make crosses to put out of the doors during St. John’s night**	
				Mix: twigs used to stir milk to curdle it [37]	
			Whole plant	Sup/rel: plant has protective value	
				Prov: ‘*Anno ficaio, poco granaio*’, ‘*Non vale un fico secco*’	
*Foeniculum vulgare* Mill*.*	*Apiaceae*	*Finocchio selvatico (c, o, g), finocchio cavallì (c), finocchietto (g)*	Roots	Med: root infusion as diuretic [37]	
			Seeds	Med: seed infusion as galactagogue [23], digestive [25], as anti-anaemic [23], to heal colics	
				Food: to flavour bread [37]	
			Leaves, seeds	Food: to flavour pork, suckling pig (‘*porchetta*’), rabbit, sea and land snails, olives, for boiling chestnut [4, 21, 23, 30, 37, 39, 42, 44, 45]	
				Vet: **leaves put in cattle feed to heal abdominal bloating**	Similar use of leaves for food use [37]
			Flowers	Food: to flavour baked mushrooms, olives [37]	
*Fragaria vesca* L*.*	*Rosaceae*	*Fragola selvatica, fragolina di bosco (g)*	Fruits	Food: fruit eaten as fresh fruit or in jams [37, 44]	
*Fraxinus ornus* L*.*	*Oleaceae*	*Ornello*	Leaves	Food: leaves used as substitute for tea	Similar use for the fruit [37]
*Fumaria officinalis L*.	*Papaveraceae*	*Erba de purghe (o)*	Leaves	Med: leaves and aerial parts crushed and used as emollient [25]	
				Food: ***some leaves in soups***	Similar use of the ‘fruit’ [36]
				Sup/rel: **burning leaves has protective value**	
*Galium aparine* L*.*	*Rubiaceae*	*Attaccamà (o)*	Leaves, stems	Med: **leaf and stem infusions as depurative and anti-inflammatory**	
				Mix: leaves and stems used as rennet for milk	Similar use for *Galium* sp. [37]
*Gentiana lutea* L*.*	*Gentianaceae*		Roots	Food: roots notoriously used in liqueurs in the Apennine area [27, 37]	
*Geranium dissectum* L*.*	*Geraniaceae*	*Sbrandello (o)*	Leaves	Med: leaf infusion as anti-haemorrhoidal	The same use for *Geranium robertianum* L. [37]
				Dye: **dye in brown**	
*Hedera helix* L*.*	*Araliaceae*		Leaves	Med: leaf infusions as decongestant and to heal menstrual pain [37]	
				Cosm: leaf decoctions used to stain hair [21]	
				Dye: leaf decoction used to revitalise dark colour and to dye green [4, 37]	
			Whole plant	Sup/rel: plant has protective value	
*Hedysarum coronarium* L*.*	*Leguminosae*	*Lupinella (o, c, g), lupina (g)*	Leaves	Med: **leaf infusion as galactagogue**	
				Vet: leaves in feeding of livestock [37]	
			Flowers	Sup/rel: ‘*infiorata’* [23]	
			Leaves, shoots, flowers	Food: leaves and flowers raw in salad [37], boiled in vegetable mixtures [41], peeled stems eaten as snack [24]	
*Helianthus tuberosus* L*.*	*Asteraceae*	*Topinambur, girasole selvatico (g)*	Tuber	Food: boiled tubers to season risotto [39, 44]	
*Helminthotheca echioides* (L.) *Holub*	*Asteraceae*	*Speraina (c), speragne, sporagne, crispigne, grugni (g)*	Leaves	Food: basal rosette boiled alone or in vegetable mixtures as side dish, used for filling ‘*crescia*’ and ‘*piadina*’ [21, 30, 34, 39, 44]	
*Humulus lupulus* L*.*	*Cannabaceae*	*Luppero (g)*	Shoots	Food: young shoots boiled and used to make omelettes [27, 39]	
*Hypericum perforatum* L*.*	*Hypericaceae*	*Scacciadiavoli, erba di S. Giovanni (g)*	Flowers	Med: flowers in olive oil, then put in the sun, as cicatrizer, against burns [4, 21, 23, 26]	
				Food: flowers for flavouring grappa [37]	
				Dye: flowers used as yellow dye [37]	
			Aerial part	Sup/rel: in St. John’s water [37] for various ritual uses during St. John’s night (see *Artemisia vulgaris*)	
*Hypochaeris achyrophorus* L*.*	*Asteraceae*	*Cosce di vecchia (o)*	Leaves	Med: leaf infusion as diuretic	The same use for *Hypochaeris radicata* L. [37]
				Food: ***leaves boiled and used to make omelettes (‘they are sweet’)***	Similar use for *Hypochaeris radicata* [21, 34, 37]
			Whole plant	Vet: pigs eat the roots, **leaves given to cattle as galactagogue**	
*Inula conyza* (Griess.) DC.	*Asteraceae*		Stems	Rep: plants hung up in the granaries to keep rats away [27]	
*Jasminum officinale* L*.*	*Oleaceae*	*Gelsumì (o)*	Flowers	Med: **flowers decoctions to heal cough**	
				Cosm: flowers in bath water to relax [36]	
				Dom: flowers used to decorate house	
			Whole plant	Sup/rel: **plant has protective value**	
*Juglans regia* L*.*	*Juglandaceae*		Leaves	Sup/rel: some leaves put in St. John’s water [23, 36]	
			Fruits	Food: fruit eaten as dry fruit, for seasoning pasta, for flavouring bread. Fruit harvested in St. John’s night to make ‘*nocino*’ [4, 37, 42]	
			Whole plant	Sup/rel: plant has some negative effects [4, 37]	
				Prov: ‘*Noce, croce*’; ‘*Beati chi ha ‘rcacciato noce e ulive perchè non se vanga e non se zappa*’	
*Juniperus communis* L*.*	*Cupressaceae*		Fruits	Food: fruit for flavouring grappa [23]	
				Cosm: fruit chewed against halitosis	Similar to the *Juniperus oxycedrus* L. use [27]
*Juniperus oxycedrus* L*.*	*Cupressaceae*	*Ginepro (c)*	Fruits	Med: fruit chewing to heal inappetence [23]; fruit juice eaten to heal stomach acid, fruit poultice on skin to heal sores	
				Food: for flavouring roast meat, liqueurs [21, 37]	
				Vet: crushed fruit added to water as galactagogue for cattle	Used cited for *Juniperus communis* L*.* [37]
				Sup/rel: **fruit in the St. John’s water**	
*Laurus nobilis* L*.*	*Lauraceae*	*Laru (o), alloro, baccarolo (g)*	Leaves	Med: leaf infusion as digestive [21, 37]	
				Food: leaves used to flavouring meat (‘*spiedini*’, ‘*fegatelli*’, meat sauces) and fish, in boiling water of chestnuts [21, 30, 41, 42, 44, 45]	
				Cosm: leaves in bath water to relax [37]	
				Sup/rel: leaves in St. John’s water [36]	
				Rep: some leaves in pots where figs were kept to keep worms away; leaves on doors to keep cockroaches away	Similar uses [4, 21, 37]
			Twigs	Recr: twig crackling in fire	
			Whole plant	Sup/rel: plant on the house entrance protects against lightning [37]	
*Lavandula* sp.	*Lamiaceae*	*Spigonardo (o), lavanda (c, g) spighette (c), spighetto (g)*	Flowers	Med: flowers in water to clean wounds [23], flowers macerated in alcohol to heal louse; to encourage sleep in children, dried spikelets placed near beds	
				Vet: **some spikelets in feed of dairy cows to flavouring the milk**	
				Cosm: flowering tops macerated in water to perfume skin [26]	
				Sup/rel: spikelet in St. John’s water; ‘*infiorata*’ [4, 37]	
				Dom: dry spikelets into drawers to perfume clothes; in floral decorations [37]	
			Leaves	Med: fresh leaves chewed to heal gingivitis [4, 37]	
			Whole plant	Prov: ‘*Una buona raccolta vale più di un campo di grano*’	
*Leopoldia comosa* (L.) Parl.	*Asparagaceae*	*Cipollaccio (g)*	Bulbs	Food: bulbs eaten raw in salads or boiled, to make omelettes [39, 41]	
*Ligustrum vulgare* L*.*	*Oleaceae*		Twigs	Mix: twigs used to make string in the grapevines [37]	
*Linum usitatissimum* L.	*Linaceae*	*Lino coltivato*	Seeds	Med: seed poultice applied to chest as decongestant, to heal cough [23]	
				Food: **seeds for flavouring bread**	
*Lunaria annua* L*.*	*Brassicaceae*	*Erba della luna, monete del papa (o), soldi, pianta dei soldi, dollari (g)*	Leaves	Med***:*** **leaf infusion as diuretic**	
				Food: **boiled leaves in vegetable mixtures**	
			Fruits	Dom: **dried plant with siliquae used to decorate house**	
				Mix: **flowers used to make wedding bouquets**	
			Whole plant	Sup/rel: **where plant grows, there it brings richness**	
*Malus sylvestris* (L.) Mill.	*Rosaceae*	*Melette selvatiche (g)*	Fruits	Food: fruits eaten raw, cooked, in jams [37, 42]	
				Vet: wasted fruit were given to pigs	
				Prov: ‘*Dare le mele ai porci*’	
*Malva sylvestris* L.	*Malvaceae*	*Malva, malbe (c), malbe (g)*	Leaves	Med: leaf infusion as laxative [21, 30], relaxing, depurative [4], for intimate washing; chewing leaves to heal toothache [4, 22, 26]; wrap of boiled leaves to heal skin diseases [4, 26], sores; wrap of boiled leaves put on chest (with ‘*pancotto*’) to heal bronchitis [23]	
				Food: raw [30, 39] or boiled [30, 39, 41, 44] leaves in salads and vegetable mixtures; boiled leaves for seasoning risotto	
				Vet: leaf infusion to heal cattle diarrhoea and as digestive; raw leaves as feed to increase milk production in dairy cows [37]	
			Flowers	Med: flowers decoctions to heal sores [21, 26, 37]	
				Food: **flowers used to make refreshing drink**	
				Sup/rel: flowers in St. John’s water [4]	
			Stems	Med: stem used as laxative suppositories for children	
				Food: stem raw in salads	
			Whole plant	Prov: ‘*Bocca malva, scappa ortiga*’, ‘*La malva da tutti i mali salva*’	
*Matricaria chamomilla* L*.*	*Asteraceae*	*Capumilla (c)*	Flowers	Infusion: flowers infusion as sedative [4, 23], digestive, depurative [4], to heal haemorrhoids [37]; flower poultice for eye inflammation [4, 21, 23], flowers poultice put on forehead against headaches [36]	
				Food: flowers used for flavouring liqueurs [37]	
				Cosm: flowers infusion to lightening hair [4]	
				Sup/rel: **flowers used in ‘i*nfiorata*’**	
				Dye: flowers to dye wool yellow [37]	
				Recr: **necklaces and bracelets with flowers**	Similar use for *Bellis perennis* L. [37]
				Dom: **flowers to perfume drawers**	
				Prov: ‘*Il tappeto di camomilla più è calpestato e più scintilla*’	
*Medicago lupulina* L*.*	*Fabaceae*	*Erba nera (o)*	Flowers, leaves	Med: **leaf and flowers infusion as lenitive and emollient**	
				Vet: **leaves and flowers as feed for livestock**	
*Medicago sativa* L*.*	*Fabaceae*	*Erba melica (c)*	Leaves	Med**: leaf infusion as tonic**	
				Vet: leaves and flowers as feed for livestock [37]	
*Melissa officinalis* L*.*	*Lamiaceae*		Leaves and flowers	Med: leaf infusion as sedative, depurative [37]	
				Food: leaves and flowers raw in salads, for flavouring meat [30, 42]	
				Cosm: leaves and flowers in water to tone skin [37]	
				Rep: **dry leaves in drawers to kept moths away**	
*Mentha x piperita* L*.*	*Lamiaceae*		Leaves, flowers	Med: leaf infusion as depurative; leaf juice in vinegar to heal vomiting [37]; fresh leaves to heal insect bites [26, 30]	
				Food: leaves raw in salads, to make sauce for meat, risotto, syrup [4, 30, 41, 42, 44, 45]	
				Sup/rel: **some protective uses attributed to the plant**	
*Misopates orontium* (L.) Raf*.*	*Scrophulariaceae*	*Borsa del pastore, sacca del pastore (c)*	Aerial part	Food: **leaves raw in salads or boiled in vegetable mixtures**	
*Morus alba* L*.*	*Moraceae*	*Moro (g)*	Leaves	Vet: leaves to feed livestock in winter, to feed silkworms [37]	
			Flowers	Dom: **flowers use in floral decorations**	
*Morus nigra* L.	*Moraceae*	*Moro (o)*	Roots	Med: **root juice against scorpion poison**	
			Fruits	Food: raw, in jams, for flavouring grappa [4, 37, 41, 42, 45]	
				Sup/rel: **unripe fruit as amulet**	
			Leaves	Med: leaves in packs to heal skin inflammations [37]	
				Dye: plant used to dye wool yellow [37]	
*Myosotis arvensis* (L.) Hill	*Boraginaceae*	*Non ti scordar di me (o)*	Aerial part	Med: leaf packs on tired eyes	Similar to the use cited for *M. ramosissima* [37]
				Vet: **leaves to feed livestock**	
*Nigella damascena* L*.*	*Ranunculaceae*		Seeds	Food: ***seeds use to flavour bread***	Similar use for pastries [36]
			Flowers	Dom: **dry flowers in floral decorations**	
*Ocimum basilicum* L.	*Lamiaceae*		Leaves, flowers	Med: leaf and flowers infusion as sedative, galactagogue, bactericide, anti-inflammatory [27]	
				Cosm: leaves in water bath as skin tonic and purifier [26]	
				Sup/rel: dry leaves to make incense	Funeral use [37]
				Rep: plants near the windows to keep mosquitoes away [4]	
*Olea europaea* L*.*	*Oleaceae*	*Ulìo (o)*	Leaves	Med: leaf decoction as hypotensive [4, 21, 33]; packs of leaves boiled in water on chest as decongestant	
				Sup/rel: some leaves on windows to protect against hailstorms	Similar use in [32]
			Oil	Med: oil to heal burns [21, 26, 33], rheumatic pain; hot oil (heated in half eggshell on embers) to heal earache [24], hot oil for rubbing on chest against bronchitis [21, 33], hot **oil to heal calluses**	
				Vet: oil rubbed on animals that had lost hair [37]	
				Cosm: oil pack on hair	
				Dom: oil used in lamps and to make detergents and soaps [37]	
			Twigs	Sup/rel: use of oil to heal devil’s eye [37], for protective use in the field see *Arundo donax*; **twigs used in predictive ritual**	
			Wood	Dom: wood use as fire starter in oven (see *Crategus monogyna*) [37]	
			Whole plant	Prov: ‘*Il nonno la pianta, il babbo la raccoglie, il nipote ci si scalda*’	
*Origanum majorana* L*.*	*Lamiaceae*		Leaves and flowers	Med: leaf infusion to heal cough [25]; infusion in wine to heal intermittent fever	
				Food: flavouring [21, 41]	
*Origanum vulgare* L*.*	*Lamiaceae*	*Menta bastarda (o)*	Leaves and flowers	Med: leaf decoction with internal use as digestive and antispasmodic [27, 44], external use to heal lice	
				Food: flavour vegetables, pizzas [4, 23, 39, 45]	
				Sup/rel: dry leaves in pocket as necklace to protect against devil’s eye	
*Ornithogalum umbellatum* L*.*	*Asparagaceae*	*Lacrime della madonna (g)*	Whole plant	Sup/rel: **where plants grown there is protection of the Madonna**	
*Ostrya carpinifolia* Scop*.*	*Betulaceae*	*Carpino (g)*	Leaves	Med: leaves macerated as anti-catarrhal	
				Vet: leaves as feed for livestock [37]	
			Wood	Craft: handles, tools [4, 37]	
*Pallenis spinosa* (L.) Cass.	*Asteraceae*		Whole plant	Mix: in the garden, as decorative	
*Papaver rhoeas* L*.*	*Papaveraceae*	*Rosoletta, rosolaccio (o), papola (c), papatelle, papaverella (g)*	Leaves	Med: cooking water as depurative	
				Food: basal rosette boiled in vegetable mixtures, as seasoning for polenta [4, 21, 34, 39, 41, 42]	
				Vet: leaves as feed for hens to increase egg laying [31]	
			Seeds	Food: for flavouring bread	
			Flower	Med: flower infusion to enhance sleep [4, 21], in enema to heal haemorrhoids	
				Cosm: petals used for make-up [26]	
				Sup/rel: flowers used in ‘*infiorata*’ [4]	
				Recr: children played guess the colour of the still closed flower: white, pink or red, saying ‘*frate, monaca o cappuccino*?’ (monk, nun, or Capuchin?) [4]; flowers used to make ‘*ballerine*’ (dancers) by folding down petals and tieing them with blade of grass; calyx used to make stamps for the skin	
			Whole plant	Prov: ‘*Il rosso del campo è la vergogna del contadino*’	
*Parietaria officinalis* L*.*	*Urticaceae*	*Erba murale, erba vetriola (c), erba vitriola (g)*	Leaves, aerial part	Med: crushed leaves to heal bruises [23, 26], leaf infusion as diuretic [4], fresh leaves to heal bites, burns, furuncles [4, 21, 26, 36]	
				Food: leaves boiled in vegetable mixtures, as seasoning for pasta, in soups (also with *Urtica dioica* L. leaves) [34, 37, 44]	
				Dom: plant used to clean flasks/bottles [4]	
*Passiflora caerulea* L*.*	*Passifloraceae*		Fruits	Food: **food eaten as fresh fruit**	
			Flowers	Dom: **flowers used in floral decorations**	
*Pastinaca sativa* L. subsp. *urens* (Req*.* ex Godr.) Celak*.*	*Apiaceae*	*Erba sellerina (g)*	Whole plant	Rep: plants **left to grow near orchards to keep thieves away**	
*Pelargonium* sp.	*Geraniaceae*		Whole plant	Rep: used to put some plants on the window sill to keep mosquitoes away	
*Petroselinum crispum* (Mill.) Fuss	*Apiaceae*	*Erbetta (o, g)*	Leaves	Med: crushed leaves to heal insect bites [4, 31]; leaf infusion or eat large amount of leaves to abort [37, 43]; leaf infusion on the skin to heal sunburn	
				Cosm: leaf infusions for lightening skin spots	
			Seeds	Med: seed infusions as diuretic [37]	
			Whole plant	Sup/rel: **plant has negative effects and predictive uses**	
				Prov: ‘*Stare in mezzo come il prezzemolo*’	
*Phaseolus vulgaris* L*.*	*Fabaceae*		Seed	Med: seed decoctions as diuretic, anti-diabetic, anti-hypertensive [27]	
				Sup/rel: **dried beans as good-luck amulet**	
*Picris hieracioides* Sibth. and Sm*.*	*Asteraceae*		Leaves	Med: cooking water as diuretic	
				Food: leaves boiled in vegetable mixtures as side dish [4, 21, 30, 34, 39]	
*Pimpinella anisum* L*.*	*Apiaceae*		Seeds	Med: seed infusion as galactagogue [36]; antispasmodic [37]	
				Food: seeds commonly used in Marche region to make liquors [23]	
*Pinus pinea* L*.*	*Pinaceae*		Young cones, buds	Med: buds infusion to heal respiratory affections [21, 37]	
			Seed	Food: seeds for seasoning pasta, to make cakes	
			Bark	Dye: bark used by fishermen to dye their fishing nets red [24, 36]	
			Pitch	Cosm: **pitch used to make sort of hair spray**	
				Mix: **resin used to make turpentine**	
*Plantago lanceolata* L*.*	*Plantaginaceae*	*Lingua di cane (o, c), orecchie di pecora (o), recchie d’asino, recchiole (c), orecchie di pe’, centonervi (g)*	Leaves	Med: leaf infusion as anti-diarrhoeal; leaf packs to heal insects bites [4, 21, 33] and sprains [4, 31], as haemostatic	
				Food: raw leaves in salads, boiled leaves in vegetable mixtures, in soups [39, 48]	
				Vet: leaves as feed for hens and rabbits [4, 21]	
				Dye: **leaves to dye clothes green**	
			Ears, stems	Recr: kids competed for those who throw the ear farthest away: stems used to make cricket cages [4]	
*Plantago major* L*.*	*Plantaginaceae*		Leaves	Food: boiled leaves in vegetable mixtures [39, 48]	
*Polygonum aviculare* L*.*	*Polygonaceae*	*Erba dei centonodi (c)*	Stems	Mix: **stems used to make ties**	
*Populus alba* L*.*	*Salicaceae*		Twigs	Vet: **young dried twigs given to rabbits and sheep in winter**	
*Portulaca oleracea* L*.*	*Portulacaceae*	*Sportellacchia, porcellana (c), erba grassa, procacchia, procaccia (g)*	Leaves	Med: fresh leaves chewed to heal gingival inflammation; crushed leaves to heal pimples [30, 43]	
				Food: raw leaves in salads, soups; boiled leaves pickled in vinegar [4, 34, 36, 39, 42, 44, 48]	
*Primula vulgaris* Huds*.*	*Primulaceae*		Leaves, flowers	Food: raw leaves and flowers in salads [39]	
*Prunus avium* (L.) L*.*	*Rosaceae*	*Cerase Selvatiche, cerase (g)*	Fruits, peduncles	Med: peduncles infusion as depurative and laxative [37]	
				Food: fruit eaten as fresh fruit	
			Leaves	Cosm: leaf infusion to rehydrate skin	
				Rep: some to keep fleas away from hen-house [29]	
			Wood	Dom: **wood used as light starter**	
			Whole plant	Sup/rel: **predictive value attributed to plant**	
*Prunus cerasus* L*.*	*Rosaceae*	*Visciola (g)*	Fruits	Food: fruit put under sugar and commonly used to make ‘*vino di visciola*’ (sour cherry wine) [37]	
*Prunus dulcis* (Mill.) D.A.Webb	*Rosaceae*		Leaves	Med: leaves and epicarp decoction to heal cough [26, 36, 37]	
				Sup/rel: **predictive value attributed to plant**	
*Prunus spinosa* L*.*	*Rosaceae*	*Prugnolo, brugnolo (c, g), scancio (g)*	Fruits	Med: cooked fruit as anti-diarrhoeal [30]	
				Food: raw fruit eaten as snack (only after first frost period); to make jams, liqueurs	
*Pulicaria dysenterica* (L.) Gaertn*.*	*Asteraceae*	*Mentastro (o)*	Aerial part	Med: plant infusion as anti-diarrhoeal [37]	
				Rep: plants burned in the hen-house to kill parasites [37]	
*Punica granatum* L.	*Lythraceae*		Fruits	Med: fruit were eaten raw to heal diarrhoea or heated with honey to heal cough [37]	
				Food: fruit eaten raw [41, 42]	
				Sup/rel: **fruit were used in a propitiatory ritual**	
*Quercus ilex* L*.*	*Fagaceae*	*Elce (o)*	Acorns, bark	Med: decoction as anti-diarrhoeal and anti-inflammatory [37]	
			Acorns	Food: roasted acorns as a surrogate for coffee, milled acorns to make bread [5, 37]	
				Vet: acorns to feed pigs [37]	
*Quercus pubescens* Willd*.*	*Fagaceae*	*Quercia*, *cerqua (g)*	Leaves	Med: leaves smoked against malaria	
				Mix: dried leaves of *Quercus pubescens* as tobacco substitutes [37]	
			Acorns	Vet: acorns to feed pigs: to prepare mash (‘*berò*’) with barley, corns, and water; rabbits: as medicinal feed for rabbits with diarrhoea [23, 37]	
			Galls	Recr: **galls used as marbles**	
			Whole plant	Prov: ‘*La cerqua ha fatto sempre la ghianda*’, ‘*Se u primu de maggio me gela i pia, poca ghianda magna u porcu mia*’	
*Quercus robur* L*.*	*Fagaceae*	*Quercia, midullo (g)*	Acorns	Vet: acorns to feed pigs [37]	
				Recr: **half cut acorns used as dolls ‘eyes**	
			Galls	Recr: **galls were used as marbles**	
			Wood	Craft: wood used to make various tools and furniture, to make kneading tables, manger (‘*greppia*’) for livestock	Similar uses referred to *Quercus* sp., [37]
*Ranunculus bulbosus* L*.*	*Ranunculaceae*	*Bottoncino d’oro (g)*	Leaves	Med: fresh leaves to heal cold sores	Similar use for *Ranunculus velutinus* Ten*.* [26, 37]
*Ranunculus velutinus* Ten*.*	*Ranunculaceae*		Leaves	Med: crushed leaved in packs to heal sciatica	Similar use for *Ranunculus bulbosus* L. [37]
				Food: ***leaves boiled in vegetable mixtures***	Similar use for *Ranunculus bulbosus* L. [37]
*Raphanus raphanistrum* L*.*	*Brassicaceae*	*Senapi (c)*	Leaves	Food: leaves boiled in vegetable mixtures [4, 21, 39, 41, 44]	
*Reichardia picroides* (L.) Roth	*Asteraceae*	*Caccialepre (c, g), scaccialepre, caccialè (g)*	Leaves	Med: leaves eaten or in infusion as depurative [21, 37]; refreshing [37], diuretic, analgesic, anti-scorbutic; fresh crushed leaves to heal toothache and headache [43]	
				Food: leaves raw in salads, boiled in vegetable mixtures [4, 21, 30, 34, 37, 39, 41, 44]	
*Robinia pseudoacacia* L.	*Fabaceae*	*Scarpette della madonna (o), cascia (g)*	Flowers	Med: flowers decoction sedative [30]	
				Food: flowers fried in sweet batters; for flavouring grappa [4, 30, 42, 45]	
				Sup/rel: flowers used in St. John’s water; in ‘*infiorata*’ [4, 37]	
				Mix: **flowers used in floral decorations in churches**	
			Leaves	Vet: some leaves for feeding rabbits (‘for other animals they are poisonous’)	Leaves in fodder [37]
			Seeds	Sup/rel: **dried seeds used to make rosaries**	
			Roots	Mix: **roots used to make ties**	
			Wood	Dom: wood used as firewood [37]	
*Rosa canina* L*.*	*Rosaceae*	*Rosa selvatica (c, o, g), rosa di macchia (o)*	Fruits (pseudo-fruits), without internal hair	Med: **fruit infusion as febrifuge**	
				Food: fruit used to make jams (sometimes with apples) [4, 44]	
				Vet: **fruit for feeding hens**	
				Cosm: crushed fruit as beauty mask	
				Recr: fruit to make necklaces [37]	
			Leaves	Med: **fresh leaf infusion to heal wounds, as cicatrizer**	
			Flowers	Med: petals macerated in vinegar to heal insect bites; petal infusion as laxative, diuretic [37]	
				Food: petals used to make liquors [37]	
				Sup/rel: flowers used in St. John’s water; ‘*infiorata*’ [4]	
				Cosm: petals in infusion for a month in water to make water rose [26]	
				Dom: **perfume for the house**	
*Rosmarinus officinalis* L*.*	*Lamiaceae*		Leaves, flowers	Med: leaf infusion with wine and honey as tonic [4, 25, 30]; leaf decoction as digestive [21, 42]; leaf and flowers pack as cicatrizer; plant was smelled as tonic	
				Food: leaves and flowers for flavouring, for filling ravioli [30, 41, 42, 44, 45]	
				Vet: **some leaves for feeding dairy cattle to flavour their milk**	
				Cosm: leaf decoction to shine hair; in bath water and in ointments as skin tonic [26]	
				Sup/rel: plant has predictive value; for protective use on St. John’s night, see *Artemisia vulgaris*	
*Rubus ulmifolius* Schott	*Rosaceae*	*Spino, more (g)*	Leaves	Med: leaves decoction to heal oral cavity inflammations [4, 25]	
			Fruits	Food: fruit eaten raw, for making jams (sometimes with strawberries), for flavouring grappa [4, 41, 44]	
			Whole plants	Prov: ‘*Il rovo dice < Nella terra meglio io covo>*’ [4]	
*Rumex obtusifolius* L.	*Polygonaceae*	*Rombice (o, g)*	Roots	Med: **root decoction as tonic**	
			Leaves	Med: leaf pack to heal burns [21]	
				Food: boiled leaves in vegetable mixtures [39]	
*Rumex pulcher* L*.*	*Polygonaceae*		Roots, leaves	Med: roots and leaf decoction as anti-diarrhoeal	Similar use for *Rumex crispus* L. [37]
			Leaves	Vet: for feeding livestock [21]	
*Ruscus aculeatus* L*.*	*Asparagaceae*	*Piccasorci (g)*	Shoots	Food: boiled young shoots to make omelettes [4, 24, 41, 44, 45]	
*Ruta graveolens* L*.*	*Rutaceae*		Leaves	Med: plant sniffed as vermifuge [4, 23]; a leaf a day eaten to strengthening eyesight [37]; raw leaves eaten to heal stomach ache; pack with leaf decoction to heal tired eyes [4]	
				Food: some raw leaves in salads [23], for flavouring meat, fish, liqueurs	
				Vet: plant can cause intestinal problems for cattle	
				Sup/rel: leaf in the pocket has protective use; for protective use on St. John’s night, see *Artemisia vulgaris*	
				Rep: some plants planted near orchard to keep parasites and rats away [21, 23]	
				Prov: ‘*La ruta fa venir la vista acuta*’	
*Salix alba* L*.*	*Salicaceae*	*Moia (g)*	Twigs	Mix: twigs used to make ties and baskets [23]	
*Salix viminalis* L*.*	*Salicaceae*	*Vimini, vengo (c), vimine, vincio (g)*	Twigs	Mix: twigs used to make ties [37]	
*Salsola soda* L*.*	*Amaranthaceae*	*Roscani (o)*	Leaves	Med: **raw leaves or in decoction as depurative and refreshing**	
				Food: boiled leaves as side dish	
*Salvia officinalis* L*.*	*Lamiaceae*		Leaves	Med: leaf infusion is used as stomachic [27, 36], digestive [21], hypotensive [21], to heal diarrhoea	
				Food: raw leaves flavouring meat, fried [4, 21, 37, 41]	
				Vet: **leaves as feed for dairy cattle for flavouring their milk**	
				Sup/rel: plant related to some magic rituals	
				Cosm: fresh leaf rubbed on teeth as whitening, for refreshing breath [4, 26, 37]	
				Dom: dried leaves to perfume linen	
				Prov: ‘*La salvia salva*’	
*Salvia verbenaca* L*.*	*Lamiaceae*	*Salvia selvatica (o, g), betonica, bettonica, brettonica, vettonica (c)*	Leaves	Med: crushed fresh leaves to heal wounds [21, 31], as cicatrizer [27], dried leaves smoked to heal headache; leaf infusion with honey and lemon as digestive	
				Cosm: fresh leaves rubbed on teeth as whitening	Similar use for *Salvia officinalis* L. [37, 26, 4,]; as toothpaste [37]
				Dye: **leaves used as yellow dye**	
			Whole plant	Sup/rel: plant used as protective against devils eye [37]	
				Prov: ‘*Sa più cose della Bettonica*’	
*Sambucus nigra* L*.*	*Adoxaceae*	*Albero delle streghe (o)*	Flowers	Med: flowers infusion to heal cough [21, 27, 33, 37]	
				Food: flowers fried in sweet batter [4, 30]	
				Dom: for ripening apples, they were alternated with elder flowers [37]	
			Leaves	Med: boiled leaves to heal abscesses [4, 21, 31]	
				Rep: leaf decoction to keep ants away [36]	
			Shoots	Cosm: shoots put in olive oil and exposed to sun to make cream for chapped hands	Similar use with medulla [25]
			Fruits	Vet: **crushed fruit infusion used to improve colour of cow tails**	
				Dye: fruit used to dye clothes blue and violet, in boiling water [37]	
				Mix: crushed fruit boiled in vinegar to make ink [37]	
			Wood	Craft: to make handles, tools [37]	
				Recr: empty wood used to make blowguns [4]	
			Whole plant	Sup/rel: thought that plant had seven virtues, so it had to be respected by bowing seven times in front of it [37]	
				Prov: ‘*Spogliati quando il sambuco si veste*’ [4]	
*Sanguisorba minor* Scop*.*	*Rosaceae*		Leaves	Med: leaf infusion as anti-diarrhoeal* [25, 37], to heal wounds and burns	
				Food: raw leaves in salads [4, 30, 34, 39, 42]	
				Vet: leaves as galactagogue feed for livestock [37]	
				Prov: ‘*L’insalata non è bella se non c’è la pimpinella*’ [4]	
*Saponaria officinalis* L*.*	*Caryophyllaceae*		Aerial part	Cosm: leaf decoction to wash hair [37]	
*Satureja montana* L.	*Lamiaceae*		Leaves	Med: leaf infusion to heal oral cavity inflammation [21, 37]	
				Food: for flavouring meat, omelettes [21, 37], **vinegar**	
*Scabiosa columbaria* L*.*	*Caprifoliaceae*	*Erba di campo (g)*	Leaves	Food: boiled basal rosette as individual side dish [39]	
*Silene latifolia* subsp*. alba* (Mill.) Greuter and Burdet	*Caryophyllaceae*	*Boccon di pecora (o)*	Leaves	Food: boiled leaves (with corn cake) [37, 39]	
				Vet: **some leaves in livestock feed**	
*Silene vulgaris* (Moench) Garcke	*Caryophyllaceae*	*Consigli, colcigli (g)*	Leaves	Food: boiled leaves as individual side dish for risotto, omelettes [24]	
			Flowers	Recr: children played to make flower burst to produce biggest noise [37]	
*Sinapis alba* L*.*	*Brassicaceae*	*Rapetta (o, g), rapacciola (g)*	Seed	Med: poultice of seeds as anti-rheumatic	
				Food: **to flavour apricots in vinegar**	
				Prov: ‘*Far venire al senape al naso*’	
			Leaves	Food: raw leaves in salads [39, 41, 44, 48]	
				Vet: some leaves in livestock feed [37]	
*Solanum tuberosum* L.	*Solanaceae*		Tuber	Med: some slices as emollient to heal burns [23]	
*Sonchus arvensis* L*.*	*Asteraceae*	*Grespigno (c)*	Leaves	Food: basal rosette raw in salads or boiled in vegetable mixtures [37, 39, 42]	
				Vet: leaves as galactagogue for rabbits	
*Sonchus asper* (L.) Hill	*Asteraceae*	*Grespigna, grispigna (o), crispigne, grispigne, grespigne (g)*	Leaves	Med: leaves as galactagogue [37]	
				Food: boiled leaves in vegetable mixtures, soups, for filling ravioli [34, 39, 41, 42, 44]	
			Roots	Food**: roasted roots used as substitute for coffee**	
*Sonchus oleraceus* (L.) L.	*Asteraceae*		Leaves	Med: leaf cooking water as diuretic [27]; leaf decoctions to heal kidney stones [25]	
				Food: boiled leaves in vegetable mixtures [4, 34, 39, 41, 44]	
*Sorbus domestica* L*.*	*Rosaceae*	*Sorbo*, *sorba (g)*	Fruits	Med: **fruit decoctions as blood depurative**	
				Food: raw fruits, for jams [37, 41, 42, 45]	
*Spartium junceum* L.	*Fabaceae*		Flowers	Sup/rel: flowers in St. John’s water; in ‘*infiorata*’ [4, 23]	
				Vet: crushed flowers against parasites in livestock	Similar medicinal use [26]
			Stems	Mix: stem used to make ties and fibres [37]	
			Whole plant	Sup/rel: magical qualities were attributed to the plant because it resists fires	
*Stachys annua* (L.) L*.*	*Lamiaceae*	*Erba ella madonna (c)*	Leaves	Med: leaves infusion used to wash face to heal headache	Similar use for *Stachys* sp. [26]; *Stachis recta* [21]
			Whole plants	Sup/rel: plant used to protect against envy and bad luck [24]	
*Stachys officinalis* (L.) Trevisan	*Lamiaceae*		Aerial part	Dye: **plant used to dye wool yellow**	
*Tanacetum balsamita* L*.*	*Asteraceae*	*Caciarola (g)*	Leaves	Food: leaves used for flavouring omelettes [39]	
*Tanacetum parthenium* (L.) Sch. Bip*.*	*Asteraceae*	*Matrecara, erba amara (c)*	Leaves	Med: raw leaves to heal headache [38]; leaf infusion digestive [37]	
				Food: leaves to make sweet pancakes [37]	
			Flowers	Med: eat flowers or flower decoction as vermifuge [37]	
				Food: **flowers used for flavouring vinegar**	
			Whole plants	Rep: plants left grow up near granaries to keep rats away	Similar use [37]
*Taraxacum campylodes G. E. Haglund*	*Asteraceae*	*Soffione (o, c, g), pisciacane (o, c, g), dente di leone (c), cicoriella (g)*	Roots	Med: roots decoction as depurative [37], diuretic, and laxative	
				Food: roasted roots as coffee substitute [37]	
			Leaves	Food: basal rosettes raw in salads, boiled in vegetable mixtures as side dishes [4, 34, 37, 39, 41, 42, 44]	
				Vet: leaves as feeding for livestock [37], in particular for healing meteorism	
			Flowers	Recr: children express wish and blow the achens [37]	
*Thymus vulgaris* L*.*	*Lamiaceae*		Leaves	Med: leaf ointment as decongestant and expectorant [21]	
				Rep: **dried leaves as repellent for moths in drawers**	
*Tilia cordata* Mill*.*	*Malvaceae*	*Tijo (o)*	Flowers, bracts	Med: flowers and bracts infusion to heal cough [23]; in bath water as sedative for babies [37]; in pack for tired eyes	
*Tragopogon pratensis* L*.*	*Asteraceae*		Leaves	Food: young leaves boiled as individual side dishes or to make omelettes [24]	
*Trifolium pratense* L*.*	*Fabaceae*	*Pane del latte (o)*	Leaves	Med: leaf infusion as expectorant [27, 37]	
				Vet: feed for livestock [37]	
			Flowers	Food: fried flowers in salt batter	Different food uses of flowers [35, 42]
			Aerial part	Recr: **depending on where leaves are oriented, guess where the storm is coming from**	
*Trifolium repens* L*.*	*Fabaceae*		Leaves, flowers	Med: leaf infusion as anti-rheumatic [37]	Guarrera 2006
				Food: leaves and flowers sautéed with onion and potatoes as side dish; **flowers for flavouring bread**	Different food use in [35]
				Vet: feed for livestock	Similar use for *T. pratense* [37]
*Triticum turgidum* L*.*	*Poaceae*		Seeds	Med: boiled or hot wheat on skin as anti-rheumatic [37]	
			Ears	Sup/rel: **four ears as cross on St. John’s water**; take some ears into the house as good luck talisman; **stems and ears used in ‘festa del Covo’**	
*Ulmus minor* Mill*.*	*Ulmaceae*	*Olmo, olmo viscio (g)*	Leaves	Vet: leaves as winter feed for livestock (‘*la fronda*’) [24]	
			Branches, wood	Sup/rel: **branches used for ‘*forche di S. Giovanni*’ (St. John’s forks) during St. John’s day**	
				Craft: wood used to make many tools, like the stick to turn polenta [37]	
				Mix: young branches used to make ties [37]	
*Urospermum dalechampii* (L.) Scop. ex F.W.Schmidt	*Asteraceae*	*Grugno amaro, grugno (g)*	Leaves	Food: basal rosette boiled in vegetable mixtures [4, 21, 34, 39, 41], sautéed, for filling ‘*crescia*’ and ‘*piadina*’	
*Urtica dioica* L*.*	*Urticaceae*	*Urtiga (o), ortiga, erba cattiva (c), urtica (g)*	Leaves	Med: leaf infusion as depurative [27, 37]; boiled leaves in pack to heal wounds [31]; crushed leaves in the nose to stop nose bleed [37]	
				Food: boiled leaves as individual side dishes or in vegetable mixtures, for seasoning risotto, gnocchi, for filling ravioli, to make omelettes [4, 21, 30, 34, 41, 42, 44, 45]; **to make tea with peppermint**	
				Vet: leaves for feeding hens, turkeys and geese [23, 33]; to increase egg laying;**to heal digestion problem in cattle**	
				Cosm: leaf infusions to heal dandruff, to prevent hair loss, to wash oily hair [26]	
				Sup/rel: **leaves used in good luck practice**	
				Dye: plant cooking water used to dye fishnets green [37]	
				Rep: leaf decoction to keep parasites away from orchard [21, 37]	
			whole plant	Prov: “*Essere come l’erba cattiva*”	
*Valeriana officinalis* L*.*	*Caprifoliaceae*		Leaves	Food: ***raw leaves in salads***	
			Roots	Med: root macerate as sedative [37]	
			Plant	Sup/rel: plant is used to protect against devil’s eye	
*Verbena officinalis* L*.*	*Verbenaceae*	*Pianta per l’ematoma (c)*	Leaves	Med: crushed fresh leaves on bruises [25]	
*Veronica persica* Poir*.*	*Plantaginaceae*		Leaves	Food: **some raw leaves in salads**	
			Leaves, flowers	Cosm**: infusion as refreshing for the face**	
			Whole plants	Sup/rel: **had to say an Ave Maria if plant was trampled; plant use as amulet during trips**	
*Vicia faba* L*.*	*Fabaceae*		Pod	Med: pods used to heal warts with a particular ritual: warts marked with a bean without a pronounced embryo, saying ‘*Secchete fava, secchete porro*’ (dry up beans, dry up wart!), after 40 days bean was thrown into well	Magic ritual to heal wounds in [37]
				Vet: **milled beans as feed for turkeys**	
				Sup/rel: pod has predictive value [37]	
*Vicia sativa* L*.*	*Fabaceae*	*Vicia (o)*	Leaves	Med: leaf pack on bruises [37]	
				Vet: for feeding livestock [37]	
			Seeds	Food: milled pods to make bread [36]	
*Viola alba* Besser	*Violaceae*	*Violetta (g)*	Leaves	Med: leaf infusion as anti-cough [23, 43]	
				Food: ***raw leaves in salads*** [37]	
			Flowers	Food: to make jam (with apples) [48]	
*Viscum album* L*.*	*Santalaceae*		Whole plants	Sup/rel: plant with fruit is considered lucky charm during Christmas period	
*Vitis vinifera* L*.*	*Vitaceae*		Leaves	Med: leaf decoction to heal chilblains [27, 37], to heal diarrhoea; leaf pack as eye decongestant [36]	
				Mix: dried leaves as tobacco substitute [37]	
			Fruits	**Med: fruit eaten as depurative**	
			Wood	Wood: **wood used in protective ritual**	
*Wisteria sinensis* (Sims) Sweet	*Fabaceae*		Flowers	Sup/rel: flowers used in ‘*infiorata*’ [37]	
*Zea mays* L*.*	*Poaceae*	*Granturco*	Leaves	Dom: dried leaves to fill mattresses [4, 23]	
			Corncob	Recr: corncobs used for making dolls [37]	
			Corns	Vet: corns as feeding for hens [37]	
			Culm	Dom: dried culms to light the fire [37]	

The table lists all of the ethnobotanical uses found for the three survey areas of the Ancona district. The information given includes name of the species, botanical family, local names, parts used, and types of use. The ‘references for similar use’ column reports similar use or the same use for a different part of the plant. The new uses are marked in bold, while the new food uses for the Marche region are in bold italic. In the column ‘local name’, *o* indicates the local name in the Osimo area, *c* in the Conero area, and *g* in the Gola della Rossa–Frasassi area

*Med*, medicinal uses; *Food*, food uses; *Vet*, veterinary uses; *Cosm*, cosmetic uses; *Sup/rel*, superstitious/religious uses; *Dye*, dyeing uses; *Craft*, craft uses; *Recr*, recreational uses; *Dom*, domestic uses; *Prov*, local sayings and proverbs; *Rep*, repellent uses; *Mix*, miscellaneous uses

During the interviews, observations were often made in the field to identify the species used; alternatively, fresh samples of plants or their pictures were shown to the informants. Voucher specimens are stored at the ‘Herbarium Anconitanum’ (ANC) of the Department of Agricultural, Food and Environmental Sciences of the Polytechnic University of Marche (UNIVPM). Identification of the species was carried out on the basis of ‘Flora d’Italia’ [17], the updated nomenclature was based on online databases [18, 19] and the classification in botanical families was the one proposed by *Angiosperm Phylogeny Group* (2016) [20].

### Analysis of the data

The uses of plants that were revealed in the interviews were grouped into 12 categories: (1) medicinal; (2) food; (3) superstitious/religious; (4) veterinary; (5) cosmetic; (6) domestic; (7) dyeing; (8) recreational; (9) repellent; (10) craft uses (of the wood); (11) sayings and proverbs (in which the plants were mentioned); and (12) miscellaneous uses.

In more detail, these uses were defined along the following lines:

1. Medicinal uses: all uses related to treatment of human diseases, including food supplements and remedies against parasites (e.g. worms, lice).
2. Food uses: in addition to uses strictly related to human consumption, these included the use of aromatic species and those used as coffee and tea substitutes, or for the production of other drinks.
3. Superstitious/religious uses: these related to ritual practices integral to religion (e.g. the ‘infiorate*’*, production of rosaries, ‘*festa del Covo*’) or to popular beliefs for the protection of the person, the animals, the house, and the fields.
4. Veterinary uses: species used to improve the health and growth of livestock [21], to defend against pests, to provide fodder [22], and to increase the productivity of livestock and poultry (e.g. production of milk and eggs).
5. Cosmetic uses: those concerning the aesthetic care of the body (e.g. skin, teeth, hair), and those used for make-up and perfumes.
6. Domestic uses: those for the care, cleaning and freshening of the home, and plants used as fuel, to preserve foods, and for production of light.
7. Dyeing uses: plants that were used to dye fabrics or work tools, such as for the nets used by the fishermen.
8. Recreational uses: those that were used for the production of toys and for hobbies.
9. Repellent uses: the species that were used to free environments from insects, rodents, and other vermin.
10. Craft uses of the wood: artisan uses of the wood from the plants, such as for the production of tools, furniture, poles (e.g. handles for brooms), and work tools.
11. Sayings and proverbs that mentioned the plants: situations in which the plants had become part of common usage for sayings and proverbs.
12. Miscellaneous uses: all those various uses that did not fall into the previous categories, such as supports for plants, production of rope and vegetable fibre, decorative (floral) uses (excluding domestic ones), and for smoking and ink production, as examples.

### Choice of the reference bibliography

Various ethnobotanical studies were used as a reference to compare the uses defined in the three study areas in the present study. These articles concerned investigations conducted in the Marche region [23–27], and also in other areas in Central Italy [4, 28–34], and in the rest of Italy [21, 22, 35–45], and in Europe [5, 46–48].

## Results and discussion

Table 1 gives the details of the information collected, in terms of the scientific names of the species, the local names (where known), the botanical families, and the categories of use, with explanations for the modes of use. Table 1 gives the new uses in bold text, and the new food uses for the Marche region are underlined. Moreover, the bibliographic references are given for each species, with the same or similar uses indicated, and with mention of the same parts of the plants or the different parts used. The focus here instead is only on the new uses, or those that are particularly unusual.

### The flora of ethnobotanical interest in the Ancona district

In total, 195 species were recorded, as both herbaceous and woody plants for which there was at least one use of ethnobotanical interest. Of these, 184 are wild plants and 11 are cultivated, although used for purposes other than those for which they were cultivated. These 195 species belong to 60 families, among which the most represented were *Asteraceae* (13.3%), followed by *Lamiaceae* (7.2%), *Fabaceae* and *Rosaceae* (6.7%), and *Brassicaceae* (5.1%).

### The informants

In total, 120 people were interviewed (30 in the Mount Conero area, 55 in the Osimo area, 35 in the Gola della Rossa–Frasassi area): 82 were women and 38 were men, with ages 32–97 years, and a mean age overall of 75 years. Of these, 65% had only attended primary school, 13% completed lower secondary school, 18% secondary school, and 4% had a university degree. For their occupations at the time of the interview or before they retired, 25% were farmers, 25% craftsmen, 16% housewives, and 15% factory workers.

### Who gathers the plants

**Fig. 2:**
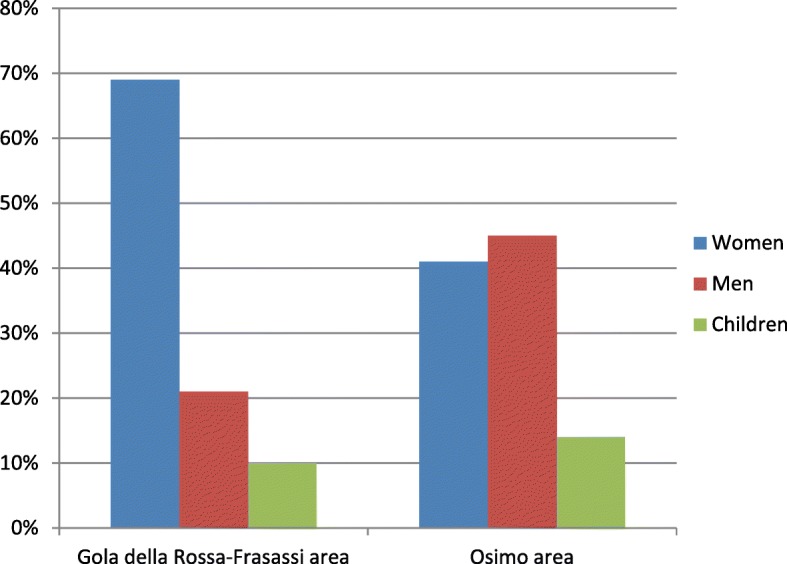
Grouping of the collectors according to age and gender for the areas of Gola della Rossa–Frasassi and Osimo

The data concerning the people who were involved in the collection of the plants were only recorded for the localities of Osimo and Gola della Rossa–Frasassi. Here, although the distributions between women, men, and children varied (Fig. 2), the gathering of the plants for the medicinal, food, superstitious/religious, domestic, and dyeing uses was the prerogative of women, while that for the wood and fruit were the task of the men. Generally, the children were mainly involved in the collection of plants for recreational uses, and sometimes for the fruit.

The oldest informant reported some particularities for the gathering of some species in the Conero area: *Matricaria chamomilla* L. could not be gathered by young boys; and those who collected *Salvia officinalis* L. had to wear a white tunic and could not use iron tools, which would have dishonoured the sacredness of the plants.

### The uses of the species of ethnobotanical interest

**Fig. 3:**
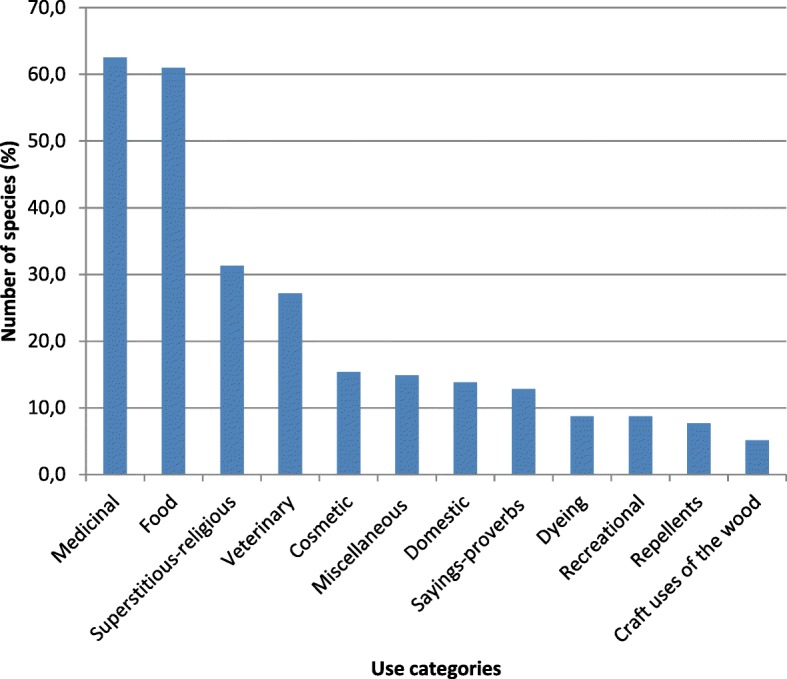
Relative numbers of the total species defined in terms of each category of use (in descending order) revealed in these three study areas

With regards to the wild plant species used in these three areas of Ancona district, the analysis shows that the species with the greatest number of categories of use here was *Sambucus nigra* L. (i.e. its use was recorded for all 12 of the categories): the flowers had medicinal and food purposes; the fruit had dyeing uses and were also used in the veterinary sector (‘to revive the colour of the tails of the cows to sell’) and to produce ink; the wood was used to produce tools; and the entire plant had superstitious/religious uses, to name just a few. Among the other species with the greatest numbers of categories of use, there were *Matricharia chamomilla* L., *Salvia officinalis* L., *Urtica dioica* L., *Papaver roheas* L., and *Rosa canina* L. (with eight categories of use each). The categories of uses and relative percentages of species are listed in Fig. 3.

#### Medicinal uses

**Fig. 4:**
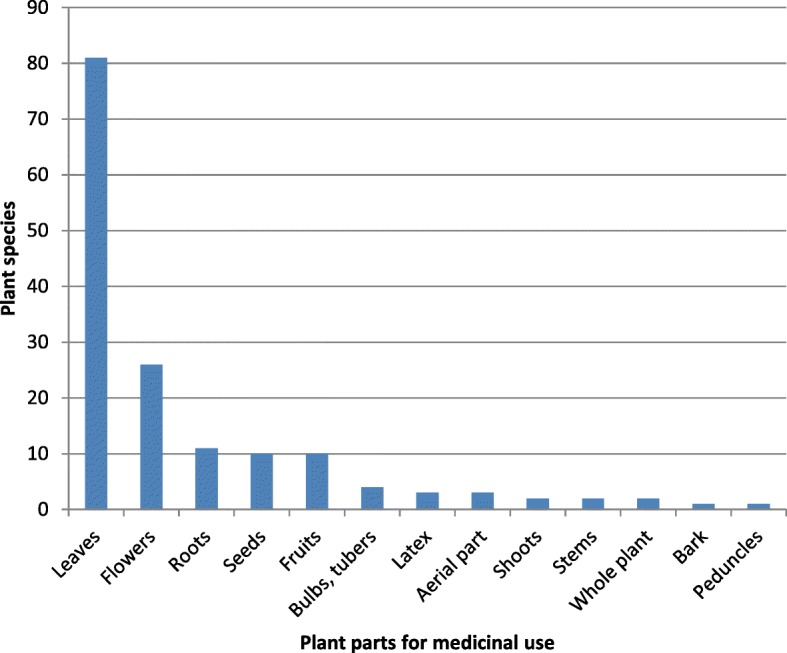
Parts of the plants used for medicinal purposes in terms of the numbers of species

Of the 195 species considered, 122 had at least one medicinal use. The most used parts of the plants were the leaves, followed by the flowers (Fig. 4).

The most common methods of medicinal use were infusions and decoctions, with the use of parts of the fresh plants. The most mentioned diseases were those that affected the skin and the gastrointestinal system, followed by those associated with the urogenital and gynaecological, respiratory, nervous, and cardiovascular systems. The species known for the same medicinal uses in all three areas were *Borago officinalis* L. against coughs; *Elymus repens* (L.) Gould. as a depurative; *Asparagus acutifolius* L. as a diuretic; *Ficus carica* L. to heal calluses; and *Malva sylvestris* L. as a laxative. The species with the highest number of different medicinal uses or used for the treatment of several different diseases were *Malva sylvestris* L. (12 different uses), *Foeniculum vulgare* Mill. (8 different uses), *Matricaria chamomilla* L., *Olea europaea* L., and *Parietaria officinalis* L. (6 different uses each).

The new uses that did not correspond to those in the literature included species used as depuratives and diuretics, like *Lunaria annua* L*.* leaves, *Salsola soda* L*.* leaves, *Galium aparine* L*.* leaves and stems, and *Sorbus domestica* L. fruit (as a blood depurative). The species used to assist digestion included leaves of *Agrimonia eupatoria* L*.*, *Diplotaxis erucoides* (L.) DC, and *Diplotaxis tenuifolia* (L.) DC. The species used as tonics were *Medicago sativa* L*.* leaves and *Rumex obtusifolius* L. roots. Other new uses were described for *Cruciata laevipes* Opiz leaves to heal intestinal obstructions, *Elymus repens* (L.) Gould to heal nose bleeds, *Hedysarum coronarium* L*.* leaves as a galactagogue (to promote lactation), and *Jasminum officinale* L*.* flowers to heal coughs. In terms of skin diseases, the new uses referred to *Medicago lupulina* L*.* leaves and flowers as an infusion as a lenitive and emollient, *Rosa canina* L*.* fresh leaves as an infusion to heal wounds, as a cicatriser, *Olea europaea L.* hot oil to heal calluses, and *Crataegus monogyna* Jacq dry fruit heated in a small bag and used to heal rheumatic pain. An unusual way to heal arthritic pain was to put crushed leaves of *Ficaria verna* Huds*.* on the skin where a blister formed and then had to be pierced. Guarrera [37] also referred the use *F. verna* as a “blistering plant”.

Some uses were instead contradictory with those given in the literature, such as *Vitis vinifera* L*.* leaves as a decoction that was previously cited as a laxative [37], while in the Gola della Rossa–Frasassi area this was used to heal diarrhoea*.* Other new uses were similar to those previously cited in the literature, but did not necessarily fully correspond to them, with the details given in Table 1.

#### Food uses

There were 119 species with food uses, some of which were used in all three study areas for the same use: *Asparagus acutifolius* L. in omelettes; *Cichorium intybus* L. as leaves in boiled vegetable mixtures; *Foeniculum vulgare* Mill. to flavour meat, fish, and olives; *Plantago lanceolata* L. in boiled or fresh vegetable mixtures; *Rosa canina* L. fruit in jams, *Salvia officinalis* L. to flavour meats; and *Urtica dioica* L. for omelettes and boiled vegetable mixtures, or for seasoning risotto. Among these, *Asparagus acutifolius* L., *Cichorium intybus* L., *Foeniculum vulgare* Mill., and *Urtica dioica* L. were the most frequently cited species for food uses across the various communities in Italy [40]. *Borago officinalis* L. and *Urtica dioica* L. were the most versatile in the kitchen, with seven different preparations.

**Fig. 5:**
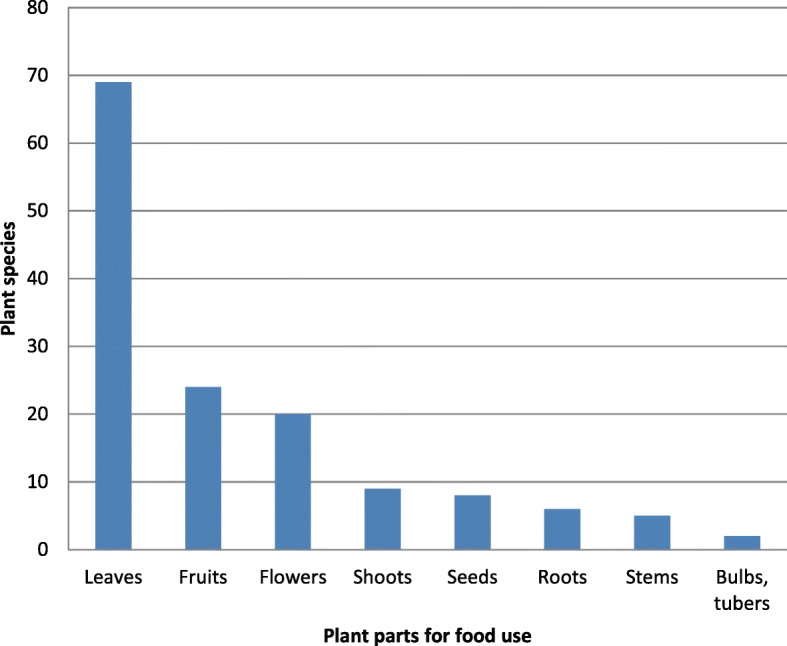
Parts of the plants used for food purposes

The most used parts were leaves, fruit, and flowers (Fig. 5), and *Foeniculum vulgare* Mill. was the species with the highest number of parts used.

Most of the species (i.e. 50 species) were boiled in vegetable mixtures, sautéed, and served as a side dish, to provide the so-called ‘foje’, which included: *Cichorium intybus* L., *Malva sylvestris* L., *Papaver rhoeas* L., *Helminthotheca echioides (L.) Holub*., *Reichardia picroides* (L.) Roth, and *Taraxacum campylodes G. E. Haglund Plantago lanceolata* L.. In some cases, these also included *Capsella bursa pastoris* (L.) Medik., *Crepis vesicaria* L., *Echium vulgare* L., *Hedysarum coronarium* L., *Plantago major* L., *Rumex obtusifolius* L., *Silene vulgaris* (Moench) Garcke, *Sonchus arvensis* L., *Sonchus oleraceus* (L.) L., and *Urospermum dalechampii* (L.) Scop. ex F. W. Schmidt.

Across the three study areas, *Crithmum maritimum* L. was only present on the rocky coasts of Mount Conero, where it was widely known: its food use as a “very delicious side dish” has already been documented by Guarrera [24]. The new uses that did not correspond to the literature consulted included some plants that were boiled in vegetable mixtures and served as side dishes, including *Lunaria annua* L. and *Misopates orontium* (L.) Raf., which was also used in fresh salads. The species that showed new uses in salads were *Veronica persica* Poir, and rhizomes of *Cynodon dactylon* (L.) Pers. as raw in salads (as collected for the Gola della Rossa–Frasassi area); these uses have only been referred to for Spain in famine periods [5].

Other new uses included fried flowers of *Achillea collina* (Beckser ex Rchb.f.) Heimerl in salted batter. In the literature, the use of this plant as fritters was reported in Sardinia [36], the use of flowers of *A. ptarmica* L. in salads for the Bologna area [42], leaves of *Agrimonia eupatoria* L. for filling fresh pasta, *Calendula officinalis* L. flowers in risotto, fruit of *Celtis australis* L. to flavour grappa, flowers of *Convolvulus arvensis* L. sucked as a snack, seeds of *Elymus repens* (L.) Gould and *Linum usitatissimum* L. for making bread, fruit of *Passiflora caerulea* L. eaten as fresh fruit, seeds of *Sinapis alba* L. to flavour pickled peaches, flowers of *Tanacetum parthenium* (L.) Sch. Bip. and *Satureja montana* L. for flavouring vinegar, flowers of *Trifolium repens* L. to flavour bread, leaves of *Urtica dioica* L. to make a tea, and *Mentha* sp., and *Malva sylvestris* L. flowers to make a refreshing drink. The pickling of leaves of *Cichorium intybus* L. (collected for Conero area) was referred to only the Mediterranean area for Cyprus [48], with roasted roots of *Sonchus asper* (L.) Hill. as a surrogate for coffee.

In addition to the new uses that are written in bold in Table 1, the new foods for the Marche region are underlined.

#### Superstitious/religious uses

Across the three study areas, 61 species had one or more superstitious/religious property. Many uses were already known in the literature for the Marche region and for the rest of Italy. However, other uses were not well described, and so it was difficult to find any correspondence with the literature.

Various uses were connected with the festivities of San Giovanni on 24 June, among which many were new. The preparation of ‘Acqua di San Giovanni’ (St. John’s water) was frequently cited, and it consisted of putting some vegetable parts and flowers in a basin of water, which was then left outside during the night of St. John (between 23 and 24 June). This water was then used to wash the face the following morning (with some reports indicating before dawn), to be free from the evil eye. Further, according to some informants in the area of the Gola della Rossa–Frasassi, the same water was thrown in a cross along the stairs and in the rooms. The species used were flowers of *Hypericum perforatum* L., *Robina pseudoacacia* L., *Lavandula* sp., *Malva sylvestris* L., *Rosa canina* L., and *Spartium juniceum* L.; leaves of *Laurus nobilis* L. and *Juglans regia* L.; shoots of *Ficus carica* L; and as a new use, berries of *Juniperus oxycedrus* L. Some of the informants indicated the use of four ears of *Triticum turgidum* L*.* placed above the St. John’s water, and this was also a use never reported before. Other rituals not previously described for the feast of St. John for the area of the Gola della Rossa–Frasassi consisted of preparing the so-called ‘forks of St. John’, with branches of *Ulmus minor* L. that were cut and stripped of the bark to be formed like the forks used with the straw, which were then placed outside the door as a good omen for the wheat harvest. Another new use, and for the same area, was to make a cross by tying some branches of *Ficus carica* L. together that were put outside the door on the night of St. John, to protect from witches. Stems of *Artemisia vulgaris* L*.*, *Ruta graveolens* L*.*, *Rosmarinus officinalis* L., and *Lavandula* sp. were put into the pockets or under the pillow to protect against witches during the night of St. John. The stem of *Artemisia vulgaris* L. provided protection when travelling.

Some flowers were used in the so-called infiorate, where drawings were designed on the ground along the streets where the Procession for Corpus Domini passed. The species used included *Bellis perennis* L., *Calendula officinalis* L., *Lavandula* spp., *Robinia pseudoacacia* L., *Rosa canina* L., *Wisteria sinensis* (Sims) Sweet, and *Hedysarum coronarium* L., with the new use recorded for *Cota tinctoria* L. J.Gay. and *Matricaria chamomilla* L.. *Triticum turgidum* L. was used (and indeed is still used) to create allegorical waggons and decorations for the ‘*Festa del covo*’, a religious celebration that is held in August in the Osimo area.

Some of the other new uses with no correspondence in the literature consulted included to keep the aerial parts of *Achillea collina* (Becker ex Rchb.f.) Heimerl in the pockets to protect against haemorrhoids. Conversely, plants of *Arum italicum* Mill. that grew near the house were weeded out to remove them, because the spots on the leaves were correlated to the blood of Jesus and the plant was believed to bring bad luck. Furthermore, some branches of *Olea europaea* L*.* were held in the hand to find lost things, with a prayer to Sant’Antonio (‘*Sacre Sponzole*’) recited. Also, a large stock of *Vitis vinifera* L*.* wood was burnt on the fire on Christmas Eve, and then allowed to burn slowly every day until 6 January, with the still-burning logs placed in the vineyard while reciting the phrase ‘*Vita mia non te ‘rrugà, t’ho portato u ceppu de Natà*’ (‘Oh my grapevine, don’t perish, I have brought you the Christmas log’).

The religious uses also included the production of rosaries, and the new plant use here was for seeds of *Robinia pseudoacacia* L.

#### Veterinary uses

Across the three study areas, 53 species had a veterinary use. Among the species that were administered as feed, new uses included *Ailanthus altissima* (Mill.) Swingle leaves, for feeding silkworms; leaves of *Celtis australis* L., for cattle; *Medicago lupulina* L*.*, *Myosotis arvensis* (L.) Hill and *Silene latifolia* subsp. *alba* (Mill.) Greuter and Burdet leaves for various animals; *Populus alba* L. dried leaves as winter feed for rabbits and sheep; and *Rosa canina* L. fruit to feed hens.

Other species were administered as curative feed, with the new uses including *Cichorium intybus* L*.* leaves, to heal intestinal worms in rabbits; and *Hypochaeris achyrophorus* L. roots, as feed for pigs and leaves for cattle, as a galactagogue. An unusual use of the bulb *Allium neapolitanum* Cirillo was to macerate it in wine to heal rabies in dogs.

Some species were used as external curatives, such as *Crataegus monogyna* Jacq*.* fruit as a poultice was used to heal ‘*spallone*’ in cattle (bruising caused by the ‘giogo’-yoke). In the literature consulted, many other species were used to heal these kinds of diseases [37]. Some species were given as feed for dairy cattle to flavour their milk, including *Alliaria petiolata* (M.Bieb.) Cavara and Grande leaves, and *Lavandula* spp., *Rosmarinus officinalis* L., and *Salvia officinalis* L. A particular use in the Gola della Rossa–Frasassi area was to dye the tails of the cows to be brought to the market, to make them brighter, using an infusion of *Sambucus nigra* L. fruit.

#### Cosmetic uses

The cosmetic uses recorded included 30 species. Among the uses in terms of hair care, one that was new and particular related to lacquer made using *Pinus pinea* L. Here, the pitch was boiled and mixed with alcohol and then put into a spray container so that it could be sprayed on the hair as a kind of lacquer. Other new uses were for leaves of *Clinopodium nepeta* (L.) Kuntze that were chewed to heal bad breath, flowers for a decoction of *Cornus mas* L*.* to heal oily skin, and leaves and flowers as an infusion from *Veronica persica* Poir. to refresh the face. An unusual use of *Ficus carica* L*.* latex was recorded in the Osimo area, where it was used to darken the skin.

#### Domestic uses

Twenty-seven species had various domestic uses. Among these, some plants were used to light fires and for wood to feed fires. The new uses here were for *Ailanthus altissima* (Mill.) Swingle, *Ceratonia siliqua* L., and *Prunus avium* (L.) L*.*

Some other new uses also included domestic floral decorations, such as *Allium neapolitanum* Cirillo, *Morus alba* L., and *Passiflora caerulea* L. flowers in fresh floral decorations, and *Aloysia citriodora* Palau*.*, *Amaranthus retroflexus* L., and *Nigella damascena* L*.* flowers and *Lunaria annua* L*.* stems with siliquae (dried seed pods) as dried floral decorations. Then there were the species used to perfume rooms and drawers, with the new and particular use reported for *Rosa canina* L., the petals of which were infused in water together with cloves and salt, to make a solution that was sprayed over the hot stove to spread the vapour through the kitchen and freshen it. *Matricaria chamomilla* L*.* flowers were also used to perfume drawers.

Other domestic uses concerned those for producing light, for detergents, and to preserve apples.

#### Dyeing uses

Dyeing uses were reported for 17 species. The new uses that did not correspond to any in the literature consulted included *Cichorium intybus* L*.*, *Salvia verbenaca* L., and *Stachys officinalis* (L.) Trevisan to dye clothes yellow; *Cruciata laevipes* O*pi*z roots as a red dye; *Geranium dissectum L.* leaves as a brown dye; and *Plantago lanceolata* L*.* leaves as a green dye. The cooking water of *Asparagus acutifolius* L. shoots was used to dye fishing nets green (reported for the Conero area).

#### Recreational uses

In the three areas, 17 species had recreational uses. The new uses that did not correspond to any in the literature consulted included ears of *Elymus repens* (L.) Gould*.*, which children used to detach them one by one to see if a desire would come true, and acorns of *Quercus* sp., which were used for dolls’ eyes, with the galls used for marbles. Leaves of *Trifolium pratense* L*.* were used to guess where a storm was coming from, which depended on the direction in which they were oriented.

#### Repellent uses

Fifteen species were cited in the three areas for their repellent uses against parasites or other damaging pests, to prevent harm coming to garden plants, or to the house, the granaries, and other stored food. The new uses here that did not correspond to others in the literature consulted included bulbs of *Allium neapolitanum* Cirillo and leaves of *Artemisia vulgaris* L*.*, which were macerated in water to keep parasites away from the orchards; and leaves of *Melissa officinalis* L*.* and *Thymus vulgaris* L*.* put in the drawers to protect against moths. A very particular use was for some plants of *Pastinaca sativa* L. subsp*. urens* (Req*.* ex Godr.) Celak*.* that were left to grow around the orchards to keep thieves away, on the basis of their urticant (i.e. itching, stinging) effects.

#### Craft uses of wood

Ten species were cited where the wood was used to make tools for agricultural, kitchen and other work activities, and for various objects for the house and the stables, and for furniture. Among the most useful woods there were *Acer campestre* L., *Ostrya carpinifolia* Scop. and *Quercus* sp., while the use of *Ailanthus altissima* (Mill.) Swingle as wood for making various tools was new, as also for *Cornus mas* L. for making boats. Particular uses included *Arundo donax* L. stems to make ‘*mazzarello*’, a tool to support knitting pins, and *Ulmus minor* Mill. as wood to make the stick used to turn polenta.

#### Sayings and proverbs

Most proverbs and idioms in which plants or parts of plants were mentioned referred to events in the agricultural life, such as the crop phases and the seasons of the year. These included, for example, ‘ (‘Lots of figs, little wheat’: in a year where a lot of figs were produced, there would be low production of wheat). Other cases might associate a person’s behaviour with the characteristics of a given plant, such as ‘*Essere come l’erba cattiva*’ (‘To be like bad grass’), which actually referred to the nettle, which was known as ‘bad grass’ locally due to its sting. In other cases, the proverbs still summarise the uses or qualities of a plant, such as ‘*La ruta fa venì la vista acuta*’ (‘Rue improves the vision’), which refers to the use of the plant to improve the eyesight.

#### Miscellaneous uses

Twenty-nine species were classified as having miscellaneous uses, which included those that do not belong to the other categories defined here. Some of these related to species that were used to make various ropes or cords, for agricultural use and for the home. For these uses, there were *Ceratonia siliqua* L. and *Polygonum aviculare* L. stems, and *Robinia pseudoacacia* L. roots. New or unusual uses were also seen for the resin of *Pinus pinea* L. to produce turpentine and for the plants that were used for wedding bouquets, such as the flowers of *Calystegia sepium* (L.) R. Br. and *Lunaria annua* L., while the flowers of *Calepina irregularis* (Ace) Thell and *Robinia pseudoacacia* L. were used to make the bride’s bouquet.

The other uses included in this category were the species where leaves were used as tobacco substitutes, for curdling milk and to produce ink.

### Local names

Some of the local names were different across these three study areas. For example, *Plantago lanceolata* L. was called ‘*lingua di cane*’ (dog’s tongue) and ‘*orecchie di pecora’* (sheep’s ears) in Osimo, ‘*orecchie d’asino*’ (donkey’s ears) and ‘*recchiole*’ (little ears) in the Mount Conero area, and ‘*orecchie di pecora*’ (sheep’s ears) and ‘*centonervi*’ (a hundred nerves) in the Gola della Rossa–Frasassi area.

Sometimes, the same local name was indicated for different species and genera, such as ‘*grugno*’ for *Cichorium intybus* L., *Helminthotheca echioides* (L.) *Holub* and *Urospermum dalechampii* (L.) Scop. ex F.W.Schmidt; and ‘*speragna*’ for *Helminthotheca echioides (*L.*)* Holub and *Picris hieracioides* Sibth. and Sm.

The local names of the plants are given in Table 1.

## Conclusions

The surveys carried out in these three study areas in the Ancona district led to the identification of ethnobotanical uses for 195 species, 184 of which were wild and 11 were cultivated. The three areas were different in terms of their economic and phytogeographic characteristics, but all of these areas were united in that they have suffered depopulation of the countryside since the 1960s, as for the rest of the Marche region and the whole of Central Italy in general. The consequence of this has been the disintegration of rural society and the loss of traditional local knowledge.

We believe that our survey can increase our present-day knowledge of the traditional local uses of plants, which now allows us to preserve this knowledge, not only in terms of medicinal and food uses, but also for ethnobotanical aspects as a whole. Some of the uses recorded here are common to all three survey areas and are also common to other areas of Marche and Central Italy, while others appear to be particularly unusual, and even new, with no previous mention of them in the literature.

The plants that were cited for medicinal uses were most numerous. *Malva sylvestris* L., *Foeniculum vulgare* Mill., *Matricaria chamomilla* L., *Olea europaea* L., and *Parietaria officinalis* L. were best known for their curative uses across the three study areas, which is in line with the rest of Italy. However, the medicinal uses of 19 species were new.

For food uses, those most noted were *Asparagus acutifolius* L., *Cichorium intybus* L., *Foeniculum vulgare* Mill., *Plantago lanceolata* L., *Rosa canina* L., *Salvia officinalis* L., and *Urtica dioica* L. Many food uses were similar to those mentioned in the literature, while among the most unusual here was the use of raw *Cynodon dactylon* (L.) Pers. in salads (for the Gola della Rossa–Frasassi area), a use that has only been reported for Spain in periods of famine [5].

For the veterinary uses of plants, the most unusual was that to ‘revive’ the tails of cows that were due to go to the market, to make them brighter, which was provided by an infusion from the fruit of *Sambucus nigra* L.

The most diffuse superstitious/religious uses were those of St. Johns’s water and the infiorate. The high number of new superstitious/religious uses arises because only a few of the literature references consulted have referred to this type of use. Among the most significant new and unusual uses were the branches of *Ulmus minor* L. and *Ficus carica* L. that were used to make the so-called forks of St. John and the crosses to hang outside the house during the night of St. John, respectively.

Among the other new and unusual uses were the cosmetic use of the *Pinus pinea* L. pitch to make a hairspray, the domestic use of the petals of *Rosa canina* L. to produce a water with which to freshen the house, and the repellent use of the plants of *Pastinaca sativa* L. subsp. *urens* (Req. ex Godr.) Celak. to protect the garden from thieves.

In conclusion, we believe that the large and varied amount of data collected here is particularly useful for its contribution to the knowledge of how plants were used by the rural societies that were widespread throughout the Marche region until the second half of the 1960s. The uses of these plants were necessary to promote the self-sufficiency of these populations in terms of their domestic and agricultural practices, and their homecare, personal care and animal care, and for their own sustenance. At the same time, we would emphasise the need to identify more than one area within even just the Marche region (here as coastal, hilly, mountainous) to provide a more complete view of the traditional knowledge that was spread throughout the territory and to allow comparisons between such areas.
